# The role of CFA/I in adherence and toxin delivery by ETEC expressing multiple colonization factors in the human enteroid model

**DOI:** 10.1371/journal.pntd.0010638

**Published:** 2022-07-26

**Authors:** Emily M. Smith, Christen L. Grassel, Antonia Papadimas, Jennifer Foulke-Abel, Eileen M. Barry

**Affiliations:** 1 Center for Vaccine Development and Global Health, University of Maryland School of Medicine, Baltimore, Maryland, United States of America; 2 Department of Medicine, Division of Gastroenterology and Hepatology, Johns Hopkins University School of Medicine, Baltimore, Maryland, United States of America; Washington University School of Medicine, UNITED STATES

## Abstract

Enterotoxigenic *Escherichia coli* (ETEC) is a primary causative agent of diarrhea in travelers and young children in low-to-middle-income countries (LMICs). ETEC adhere to intestinal epithelia via colonization factors (CFs) and secrete heat-stable toxin (ST) and/or heat-labile toxin (LT), causing dysregulated cellular ion transport and water secretion. ETEC isolates often harbor genes encoding more than one CF that are targets as vaccine antigens. CFA/I is a major CF that is associated with ETEC that causes moderate-to-severe diarrhea and plays an important role in pathogenesis. The Global Enteric Multicenter Study finding that 78% of CFA/I-expressing ETEC also encode the minor CF CS21 prompted investigation of the combined role of these two CFs. Western blots and electron microscopy demonstrated growth media-dependent and strain-dependent differences in CFA/I and CS21 expression. The critical role of CFA/I in adherence by ETEC strains expressing CFA/I and CS21 was demonstrated using the human enteroid model and a series of CFA/I- and CS21-specific mutants. Furthermore, only anti-CFA/I antibodies inhibited adherence by global ETEC isolates expressing CFA/I and CS21. Delivery of ST and resulting cGMP secretion was measured in supernatants from infected enteroid monolayers, and strain-specific ST delivery and time-dependent cGMP production was observed. Interestingly, cGMP levels were similar across wildtype and CF-deficient strains, reflecting a limitation of this static aerobic infection model. Despite adherence by ETEC and delivery of ST, the enteroid monolayer integrity was not disrupted, as shown by the lack of decrease in transepithelial electrical resistance and the lack of IL-8 cytokines produced during infection. Taken together, these data demonstrate that targeting CFA/I in global clinical CFA/I-CS21 strains is sufficient for adherence inhibition, supporting a vaccine strategy that focuses on blocking major CFs. In addition, the human enteroid model has significant utility for the study of ETEC pathogenesis and evaluation of vaccine-induced functional antibody responses.

## Author summary

Enterotoxigenic *Escherichia coli* (ETEC) is a major cause of diarrhea in travelers and in young children in low-to-middle-income countries (LMIC). ETEC infects the small intestine using surface located adhesins called colonization factors (CFs) and delivers toxins to the intestine. These CFs are optimal targets for ETEC vaccine development for the induction of colonization-blocking responses. In the current study we investigated the role of 2 CFs, namely CFA/I and CS21 when they are simultaneously expressed in geographically diverse ETEC clinical isolates, in infection using the human enteroid model. This system is the most human relevant and sensitive model to study enteric pathogens. We determined that CFA/I was important for adherence to the human enteroid by ETEC strains expressing CFA/I and CS21. Strain-specific levels of toxin delivery were quantified but there was no difference between wildtype and CF-deficient mutant strains. This result highlights the opportunity to consider the effects of intestinal movement and oxygen levels in the model. Infected enteroids did not have disrupted cell monolayers or produce inflammatory signals. This study supports the strategy to target CFA/I in ETEC vaccine development and underscores the enteroid as a significant model system.

## Introduction

Enterotoxigenic *E*. *coli* (ETEC) is a primary causative agent of diarrhea in travelers and in young children in low-to-middle income countries (LMICs), where it causes ~84 million diarrheal episodes and 44,000 estimated deaths per year in children younger than five [1–5]. ETEC adhere to the intestinal epithelium using colonization factors (CFs) that are surface-expressed fimbrial, fibrillar, or non-fimbrial structures and secrete heat-stable toxin (ST) and/or heat-labile toxin (LT), leading to disruption of cyclic nucleotide production and ion transporter expression and function and resulting in watery diarrhea [6–9]. The Global Enteric Multicenter Study (GEMS) determined that ST-only and ST-LT-expressing ETEC were significantly associated with moderate-to-severe diarrhea (MSD) [1,10]; this study investigates ST-expressing ETEC in the human enteroid model. Intestinal cell intoxication by ST results in the increased production of cGMP intracellularly as well as the polarized secretion of cGMP in the apical and, at a higher level, in the basolateral compartment using polarized transformed cell lines and the human enteroid model [11,12]. In addition to CFs, ETEC also express other virulence factors, including EatA and EtpA, that contribute to pathogenesis and disease [13–17].

**Fig 1:**
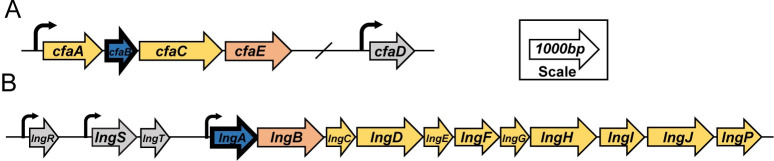
Schematic representation of the CFA/I and CS21 operons. The CFA/I (A) and the CS21 operons (B) are depicted with arrows indicating coding sequences and lines representing intergenic regions. The size of the arrows represent relative size (bp) of genes. The bolded subunits (blue) represent the major subunit of the operon. Curved arrows indicate a putative promoter.

Over 30 different types of antigenically distinct CFs have been identified in ETEC, and individual ETEC isolates can harbor single or multiple CF genes. The GEMS study identified the CFs most prevalently associated with isolates causing MSD including CFA/I and coli surface (CS) antigens CS1-CS6, which are considered major CFs [10]. CFA/I is a class 5 fimbriae encoded by a 4-gene operon and composed of repeating major structural subunit proteins (CfaB) that support one or a few CfaE subunits at the tip of the fimbriae (Fig 1). Assembled by the chaperone-usher pathway, CFA/I extends from the bacteria in a peritrichous manner, creating hair-like projections across the surface. CFA/I is one of the most well studied CFs; it has been demonstrated to be important for pathogenesis in human clinical trials as well as in laboratory-based studies and is included as a target in multiple vaccine strategies [18–22].

CS21 is a minor CF with limited data to support its contribution to ETEC-mediated diarrheal disease despite laboratory data demonstrating a role in attachment to transformed cell lines [10,23–26]. CS21, or Longus, is characterized by its laterally aggregated filaments that form a bundle of striking length over 20 μm [27]. In contrast to CFA/I, CS21 filaments extend from the cell surface in a unipolar manner [27]. CS21 shares homology to the type IV pilus, is encoded by a 14 gene cluster, and is primarily comprised of the major structural subunit LngA (Fig 1). CS21 is frequently encoded in ETEC strains in combination with other major CFs, including CFA/I; in the GEMS study, 78% of CFA/I-encoding strains also encoded CS21 [10]. This finding promoted inquiry of the potential role of CS21, particularly in a strain with multiple CFs, and for its inclusion as a vaccine antigen.

Little is known about the impact of multiple CFs on ETEC adherence and toxin delivery. Simultaneous expression of multiple CFs has been observed in clinical isolates with other major CFs, such as with CS3 or CS6 [28,29]; however, extensive expression and functional studies have not been performed using isolates with the combination CFA/I and CS21. We hypothesized that expression of CFA/I and CS21 confers increased adherence, toxin delivery, and immune evasion, and that the human enteroid model would be valuable for these studies. Previously, ETEC adherence has been studied using transformed cell lines and animal models, which lack several aspects of human disease [30]. This study takes advantage of the human enteroid model to study CF-specific roles in ETEC pathogenesis. Enteroids are derived from Lgr5+ stem cells, isolated from small intestinal crypts of healthy human donors, which can be differentiated to include multiple intestinal cell types, including goblet, Paneth, enteroendocrine, and epithelial cells [31,32]. With these specialized cell types, enteroids recapitulate the major functions of human intestine and can be seeded as monolayers on transwells to allow apical infection to understand gastrointestinal physiology and pathogenic mechanisms of a variety of microbes [12,33,34]. Enteroids have recently been used to demonstrate a role for other virulence factors, including EatA and EtpA, in ETEC adherence as well as toxin delivery [17,35,36]. The direct relevance to human biology, rigor of this system, and feasibility of use in tissue culture allow the human enteroid to serve as an invaluable and sensitive tool to study CF-specific roles during ETEC pathogenesis.

This model also allows the evaluation of the blocking capacity of anti-CF antibodies. The potential efficacy of ETEC vaccines designed to induce CF-specific colonization-blocking antibodies is supported by clinical studies that show the protective effects of administration of CF-specific antibody against WT challenge [18,37,38]. Also, CF-specific antibodies, such as those against CFA/I or CS21, are detected following natural infection [10,39,40]. There is currently no licensed ETEC vaccine but multiple strategies under development focus on major CFs [19,41–44]. However, designing a vaccine with broad coverage poses challenges due to regional differences in the diversity and prevalence of ETEC serotypes, CFs, and other important antigens [45]. This study addressed the question of whether inclusion of minor CFs in an ETEC vaccine contribute to broad protection against ETEC isolates, particularly those expressing multiple CFs. Our findings underscore the role of CFA/I in ETEC adherence across global CFA/I-CS21 ETEC isolates and support vaccine strategies that target major CFs. This work further supports the use of the human enteroid model for evaluation of adhesion-blocking anti-CF antibodies.

## Methods

### Ethics statement

Deidentified biopsy tissue from the jejunum and ileum was obtained from healthy subjects who provided written informed consent at Johns Hopkins University, and all methods were carried out in accordance with approved guidelines and regulations. All experimental protocols were approved by the Johns Hopkins University Institutional Review Board (IRB NA_00038329). All bacterial samples analyzed in these studies were de-identified and obtained from previously archived existing collections [10].

### Strains used in the study & culture media

ETEC clinical isolates and mutant strains are described in Table 1. All bacterial strains were grown on CFA agar [46] or in Terrific broth [25] for expression experiments. Strains were grown on CFA agar for electron microscopy and enteroid infection experiments, with the exception of one enteroid infection experiment using bacteria grown in Terrific broth.

**Table 1:** Bacterial strains used in this study.

Strain/Plasmid	Description (Country of origin)	Reference
H10407	LT, ST, CFA/I (Bangladesh)	[47]
H10407Kan	H10407 CFA/I^-^ (*cfaE*::Km^R^)	[21]
CVD30	ST, CFA/I, CS21 (504239, India)	[10]
EMS07	CVD30Δ*cfaB*	This study
EMS08	CVD30Δ*lngA*	This study
EMS09	CVD30Δ*cfaB*Δ*lngA*	This study
EMS10	CVD30Δ*cfaB*(pGA2-CFA/I)	This study
EMS11	CVD30Δ*cfaB*Δ*lngA*(pGA2-CFA/I)	This study
E9034A	LT, ST, CS21, CS3	[48]
*E*. *coli* pGA2-CFA/I	High level constitutive CFA/I expression	[49]
10002a	ST, CFA/I, CS21 (Chile)	[50]
100386	ST, CFA/I, CS21 (The Gambia)	[10]
102658	ST, CFA/I, CS21 (The Gambia)	[10]
200332	ST, CFA/I, CS21 (Mali)	[10]
204033	ST, CFA/I, CS21 (Mali)	[10]
300202	ST, CFA/I, CS21 (Mozambique)	[10]
302005	ST, CFA/I, CS21 (Mozambique)	[10]
401248	ST, CFA/I, CS21 (Kenya)	[10]
600609	ST, CFA/I, CS21 (Bangladesh)	[10]
702213	ST, CFA/I, CS21 (Pakistan)	[10]
702375	ST, CFA/I, CS21 (Pakistan)	[10]

### CF-specific antibodies

Anti-CFA/I and anti-CS21 antibodies were generated as a fee-for-service contract with Rockland Immunochemicals and Genscript, respectively, using purified proteins. The purified CFA/I fimbriae was purchased from BEI Resources. Purified LngA (produced by Genscript) is a recombinant peptide that is tagged with a 6X His tag. The amino acid sequence for the LngA peptide is QRAFDSRAVTDLVTNTNTVRVRMKDAYQRDGKYPDFVDPLSLTANTIKTDTSGIPAAQLVQLGKITPDEVRNNISGDFIAIGGALTSNGAQVKKGFAIELNGLSQEQCRSILGQVGNNWEYVAIGTSASGSYAMTATGVDMSVAASTTVLRSLGNNGQTTLTADKILSTCTAQVNSITLGSR (19kDa). The His-tagged protein is 21 kDa.

### Construction of mutants

The CVD30 *cfaB* and/or *lngA* mutants were engineered by allelic exchange using the lambda-red system as previously described [51], using primers and plasmids outlined in Table 2. All primers for this study are based on Genbank CP025859.1, CP025861.1, and CP025860.1. Briefly, the plasmid pKD4 and primers E01-F and E01-R (for *cfaB*) or primers E02-F and E02-R (for *lngA*) were used to amplify the kanamycin resistance gene with flanking regions homologous to *cfaB* or *lngA*. Mutants of these genes were isolated as antibiotic-resistant colonies after introduction into bacteria carrying a Red expression plasmid pKD46. The kanamycin resistance gene was then eliminated using a helper plasmid pCP20 encoding the FLP recombinase. Screening for gene deletions identified colonies with an unmarked deletion present in the target gene(s). The plasmid pGA2-CFA/I was used for trans complementation of these mutant strains [49]. Growth curves of these complemented mutants were performed in DMEM at 37°C shaking and compared to the wildtype ETEC CVD30. The OD_600_ was monitored every 30 min over time for each strain.

**Table 2:** Primers used in this study.

Primer	Description	Sequence (5’➔3’)
E01-F	pKD4-*cfaB* FWD	CATGAAGGCATAGAAAAAGAGCAAGGGCTAATACAATTAAAGGTTCCTTGATTACTCATCTATATACTAAGGAGTTCTAGTGTAGGCTGGAGCTGCTTC
E01-R	pKD4-*cfaB* REV	GCTTCATATAAATTCATTTCCATGAAAAAGGAGGGATGTATAAACATACCCCCTCCTTTTAAATAAAAGAACATATGAATATCCTCCTTA
E02-F	pKD4-*lngA* FWD	GGTTCCATGATCTTTTCAGATTGGTTGAATCAGTTGTCAGTAGATAAAATCAACAGGAGGAATACTTGTGTAGGCTGGAGCTGCTTC
E02-R	pKD4-*lngA* REV	AAAATTGTCTCTGTGAGAAGGTACTAGCCTATCATATTTAAACTAGTACCTTTTGGAAAAACATATGAATATCCTCCTTA
E03-F	Kan-*cfaB* FWD	GGGAATGTTAGAGCAGGCGT
E03-R	Kan-*cfaB* REV	CCTAGAGTTTGCCCATATAG
E04-F	Kan-*lngA* FWD	GACATGGGTTGGACCTG
E04-R	Kan-*lngA* REV	CCTGCCACTTCTGAAATC
E05-F	pKD46 FWD	TCGTTCATCCATAGTTGCCTG
E05-R	pKD46 REV	GCAGTGCTGCCATAACCATG

### Expression of colonization factors

Whole cell bacterial lysates were prepared for western blot analysis as follows. Bacteria were harvested from overnight growth on CFA agar plates or in Terrific broth static culture into PBS, and the OD_600_ was used to normalize the samples for bacterial numbers. The normalized suspensions were then mixed 1:1 with 2x Laemmli Sample Buffer (Bio-Rad). Proteins were separated on a 12% Mini-Protean TGX Precast Gel (Bio-Rad) and transferred to PVDF membrane. The membrane was blocked in a 10% (w/v) nonfat milk buffer in PBS and incubated with absorbed polyclonal Rabbit α-CFA/I (Rockland Immunochemicals) or polyclonal Rabbit α-LngA antibodies (Genscript). The secondary antibody was Goat α-Rabbit 680 nm (Invitrogen), and proteins were visualized using the LI-COR Odyssey Laser Scanner. An identical 12% Mini-Protean TGX Precast Gel (Bio-Rad) was run with each Western blot and stained with GelCode Blue Stain Reagent (Thermo Scientific) to determine the total protein in each lane. Additional Western blots of CFA agar grown and Terrific broth grown strains were performed and stained with anti-DnaK (Invitrogen) and Goat α-Rabbit 680 nm (Invitrogen) antibodies to confirm equal loading. Fiji (ImageJ) was used to quantify the amount of protein (ng) expressed by each sample within individual Western blots as compared to the protein concentration of the purified fimbrial control.

### Slide agglutination

Bacteria were harvested from overnight growth on CFA agar plates into PBS, and the OD_600_ was used to normalize bacterial numbers. Equal volumes of bacteria and anti-CF antibody were mixed, either with polyclonal anti-CFA/I (Rockland Immunochemicals) or anti-LngA (Genscript) on a microscope slide, and agglutination was observed using a light microscope. The degree of agglutination was recorded as follows [46]. A +++ reaction was an instantaneous and complete agglutination involving all bacteria, resulting in large clumps of bacteria. A ++ reaction was an instantaneous and moderate agglutination involving most bacteria, resulting in mid-large clumps of bacteria. Slower or less complete agglutination was graded +, resulting in smaller clumps of bacteria. No agglutination was graded −, indicating no clumps of bacteria.

### Electron microscopy

Transmission electron microscopy (TEM) was used for visualization of colonization factors. Bacteria grown on CFA agar were resuspended in buffer (1% BSA and 1% Tween20 in PBS) and stained with 0.5% uranyl acetate (UA, Electron Microscopy Sciences) on Formvar carbon coated copper grids 400 mesh (Electron Microscopy Sciences) before examination using Tecnai T12 TEM. Immunogold labeling of CFA/I or CS21 was performed using Formvar carbon coated nickel grids 400 mesh (Electron Microscopy Sciences). Bacterial samples were incubated in primary antibody, either rabbit anti-CFA/I (Rockland Immunochemicals) or anti-LngA (Genscript) for 1 hr. The secondary antibody consisted of 10 nm gold particles conjugated to goat anti-rabbit antibody (Sigma). The 0.5% uranyl acetate negative stain was added to grids for 1 min before visualization using TEM.

### Tissue collection and intestinal enteroid culture

Human enteroid cultures from jejunum and ileum were established from biopsy specimens obtained after endoscopic or surgical procedures by utilizing methods developed by the laboratory of Hans Clevers [32]. Four enteroid lines were used in this work: 35I, 46I, 34I, and J2. All were secretor positive as determined by immunostaining with a UEA-1 conjugate. The blood groups of the enteroid lines were also determined: 35I (type O), 46I (type O), 34I (type A), and J2 (type B). Enteroids isolated from intestinal crypt cells were cultured as 3D cysts embedded in Matrigel (Corning) and passaged approximately every 7 days. All enteroid media were prepared as reported previously [52]. 3D enteroids were harvested by incubation in an organoid harvesting solution (Cultrex) followed by vigorous shaking and trituration. To form enteroid monolayers, the triturated enteroids were resuspended and seeded on polycarbonate membrane 24-well cell culture inserts with a 0.4-μm pore size (transwell filters; Corning) that were precoated with human collagen IV solution (Sigma). Monolayers were incubated in enteroid propagation media with Y-27632 and CHIR99021 [52,53]. Typically, confluence in these enteroid cultures was achieved in 10 to 14 days as assessed by the increase in transepithelial electrical resistance (TEER) measured using an epithelial volt/ohmmeter (EVOM^2^; World Precision Instruments). Confluent monolayers were differentiated by incubation with Wnt3A-free and R-spondin-1-free medium for 5 days. Bacterial adherence and toxin delivery assays were typically performed at 5 days post differentiation. The percent change in TEER was calculated using the following formula: ((TEER_post-infection_) − (TEER_pre-infection_))/(TEER_pre-infection_) × 100%.

### Adherence and toxin delivery

Adherence assays were performed using differentiated enteroid monolayers. TEER readings of monolayers were measured at the start of the experiment and at each timepoint. Monolayers were washed with DMEM and incubated with DMEM with 1 mM IBMX (Sigma) in both apical (100 μL) and basolateral (600 μL) transwell compartments for 10min at 37°C, 5% CO_2_. Bacterial inocula were prepared in DMEM by resuspending a loopful of bacteria from CFA agar. The bacterial suspension was diluted to the desired bacterial concentration (1 × 10^7^ CFU/mL) in DMEM. For experiments with CF-specific antibodies, strains were incubated with CF-specific antibody for 1 hr at 37°C on a rotator, using anti-CFA/I (Rockland Immunochemicals) or anti-LngA (Genscript). Then 100 μL of the inoculum (∼1 × 10^6^ CFU) or specific concentration of purified ST (BEI) was added to enteroid monolayers. The infected monolayers were incubated at 37°C with 5% CO_2_ for 90 min, 4 hr, or 8 hr to allow bacterial adherence to enteroid monolayers. Following adherence, the media from apical and basolateral compartments were collected for cGMP or cytokine analysis. Monolayers were lysed with 1% Triton X-100 with 0.1M HCl to collect intracellular cGMP. Lysates and supernatants were centrifuged at 13,000xg for 5 min at room temperature to remove cell debris. Supernatants for cGMP analysis were then acidified with HCl (1:100) to inactivate phosphodiesterase activity. To isolate adherent bacteria, remaining monolayers were washed with PBS three times on the plate shaker at 550 RPM and lysed using 1% Triton X-100 by gentle scraping and incubation at room temperature for 20 min. Serial dilutions were plated in quadruplicate on LB agar and incubated overnight at 37°C. The percentage of CFU recovery was determined as follows: adherent bacteria/bacterial inoculum × 100%. For CF-antibody adherence inhibition studies, the percentage of bacterial binding inhibition was calculated using the average of wildtype bacteria enumerated as 100% binding as follows: 100% − ((no. of bacteria incubated with CF-specific antibody/no. of wildtype bacteria) × 100%) [54].

### ST production

ST production was quantified as described previously with modifications [55]. Wildtype CVD30 and H10407 strains were harvested from overnight growth on CFA agar, resuspended in DMEM at 1x10^7^ cell/mL, and incubated at 37°C with 5% CO_2_ for 8 hrs. The culture was centrifuged, and supernatant was aliquoted and frozen at -80°C until assayed for ST production using the cyclic nucleotide cGMP ELISA (Direct cGMP ELISA kit, Enzo Life Sciences). Human T84 colonic epithelial cells were cultured in DMEM-F-12 and seeded onto 24-well plates (Corning) at 5x10^5^ cells/well. Monolayers were washed with media and incubated with 1mM IBMX (Sigma) for 30 min at 37°C, 5% CO_2_. 200 μL of supernatant, 200 μL of purified 1nM ST, or 200 μL media was added to monolayers and incubated for 8 hrs. Supernatants were collected and cell monolayers were lysed with 1% Triton X-100 with 0.1M HCl to collect intracellular cGMP. Lysates and supernatants were centrifuged at 13,000xg for 5 min at room temperature to remove cell debris. Supernatants for cGMP analysis were then acidified with 12N HCl (1:100) to inactivate phosphodiesterase activity.

### Cyclic nucleotide ELISA

Cell lysates and supernatants isolated from enteroid adherence assays and T84 ST production assays were used to measure cGMP using the cGMP ELISA. Lysates and supernatants from each monolayer were divided between two ELISA wells to achieve measurements within the dynamic range of substrate detection. Well absorbance at 405nm was recorded on a Versamax microplate reader. For enteroid assays, total cGMP concentrations were calculated to absolute quantities based on the fixed volume of the enteroid lysate (200 μL) as well as in the apical (200 μL) and basolateral (600 μL) transwell compartments for a final concentration of cGMP in fmol/monolayer. For T84 assays, total cGMP concentrations were calculated to absolute quantities based on the lysate volume (500 μL) for a final concentration of cGMP in fmol. To calculate ST production in ETEC strains, background cGMP levels were subtracted from cGMP levels induced in all conditions. The amount of ST (nM) produced by the ETEC isolates was calculated relative to the amount of cGMP produced by the 1nM purified ST positive control. The amount of ST (ng) was calculated using the molecular weight of ST.

### Confocal microscopy

Enteroid monolayers were fixed using cold 4% paraformaldehyde or cold Clarke’s fixative (cold 1:1 methanol/acetic acid) and washed. Monolayers were permeabilized and blocked in a single step using PBS containing 2% bovine serum albumin (BSA), 15% fetal bovine serum (FBS), and 0.1% saponin. Immunostaining was performed with the following antibodies: anti-EpCAM (rabbit monoclonal, Abcam), anti-CFA/I (rabbit polyclonal, Rockland Immunochemicals), anti-LngA (rabbit, polyclonal, Genscript), anti-NHE3 (rabbit polyclonal, Novus Biologicals), anti-MUC2 (rabbit monoclonal, Invitrogen), anti-Lysozyme (rabbit polyclonal, Invitrogen), and/or anti-ChgA (rabbit polyclonal, Novus Biologicals). The monolayers were then incubated with Hoescht 33342 solution and Alexa Fluor-conjugated secondary antibodies (wheat germ agglutinin (WGA, Invitrogen) and F-actin (Phalloidin, Invitrogen)). The monolayers were mounted in Prolong gold antifade mountant (Invitrogen). Imaging was carried out at the Confocal Microscopy Core at the University of Maryland, Baltimore, using a Nikon W1 spinning-disk confocal microscope. Images were captured using a 40X and 60X oil objective. Image processing was completed using Fiji (ImageJ) [56].

### Cytokine ELISA

The supernatants from apical and basolateral compartments of the enteroid monolayers were collected for cytokine analysis using a DuoSet enzyme-linked immunosorbent assay (ELISA) kit for human IL-8 (R&D Systems). The amount of IL-8 is reported as picograms contained in the total volume of the culture supernatant present in the apical (200 μL) and basolateral (600 μL) compartments of the enteroid monolayers at 8 hr post infection.

### Statistical analyses

Statistical significance was determined using two-tailed, unpaired Student’s *t* test. For multiple comparisons, analysis of variance (ANOVA) was used with a Tukey’s or Bonferroni posttest to determine statistical differences within specific groups, as noted in figure legends. Each dot in the figures represents data collected from an individual monolayer. Replicates from multiple independent experiments were pooled. The number of independent experiments pooled for each figure is reported in the figure legends. GraphPad Prism software was used for all statistical analyses.

## Results

### Differential expression of CFA/I and CS21 by growth condition in wildtype clinical isolates and mutant derivative strains

**Fig 2:**
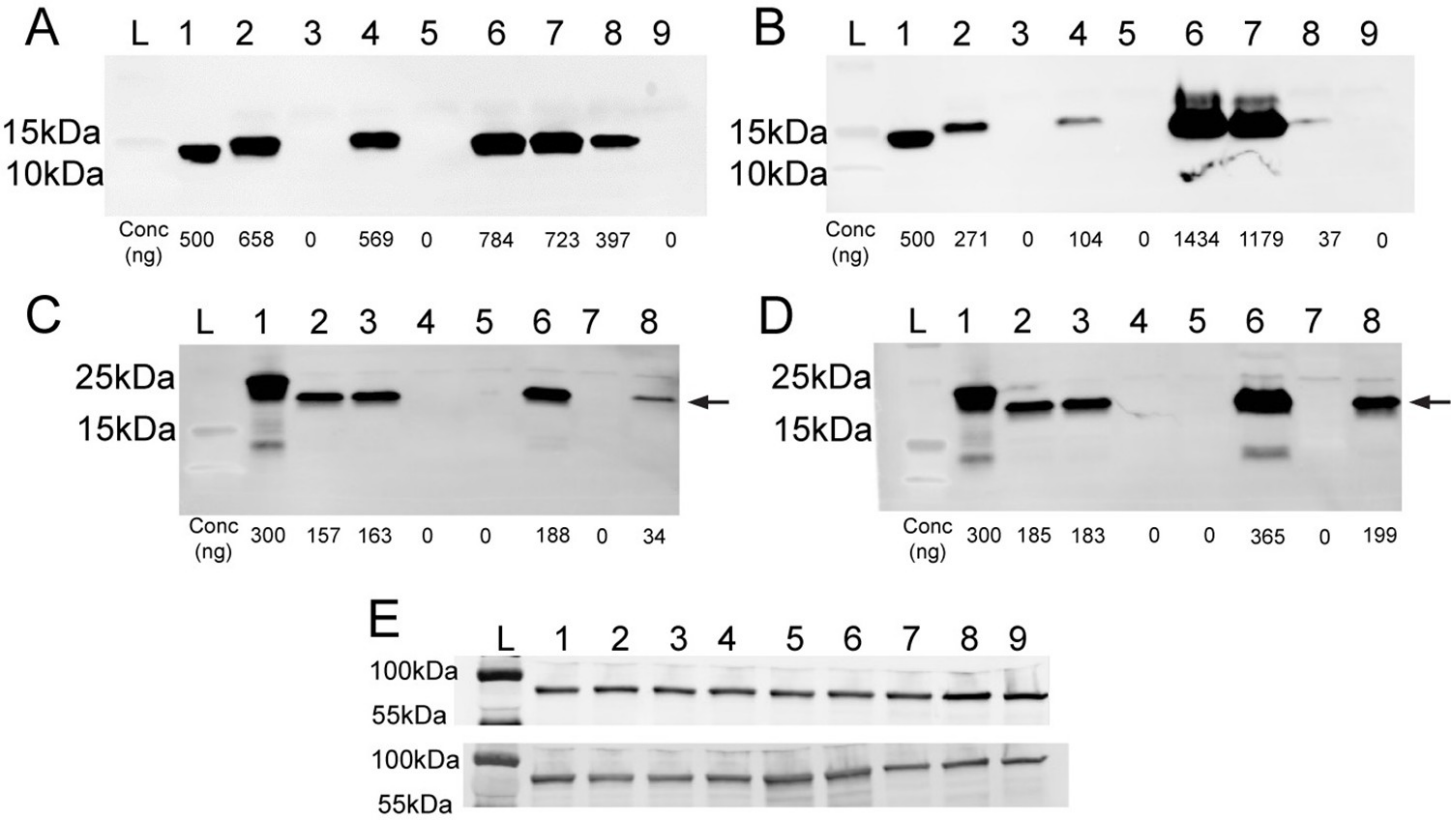
Expression of CFA/I and CS21 by ETEC strains grown in CFA agar or Terrific broth. ETEC strains were grown on CFA agar (A, C, E top) or in Terrific broth overnight static culture (B, D, E bottom). Whole cell bacterial lysates were probed with anti-CFA/I (A and B), anti-CS21 antibodies (C and D), or anti-DnaK antibodies (E). Panels A and B: ladder (L); lane 1, purified CFA/I (500ng); lane 2, CVD30; lane 3, CVD30*ΔcfaB*; lane 4, CVD30*ΔlngA*; lane 5, CVD30*ΔcfaBΔlngA*; lane 6, CVD30*ΔcfaB*(pGA2-CFA/I); lane 7, CVD30*ΔcfaBΔlngA*(pGA2-CFA/I); lane 8, H10407; and lane 9, H10407Kan. Panels C and D: ladder (L); lane 1, purified LngA (300ng); lane 2, CVD30; lane 3, CVD30*ΔcfaB*; lane 4, CVD30*ΔlngA*; lane 5, CVD30*ΔcfaBΔlngA*; lane 6, CVD30*ΔcfaB*(pGA2-CFA/I); lane 7, CVD30*ΔcfaBΔlngA*(pGA2-CFA/I); and lane 8, E9034A. Protein concentrations of CFs (ng) were determined using densitometry. Arrow indicates band size for LngA. Purified CFA/I results in a CfaB band that migrates faster than that in whole cell lysates. Purified LngA is tagged by a 6X His tag (21kDa) while LngA in whole cell lysates result in a band at 19kDa. DnaK western blots confirmed equal loading of samples. Panel E: ladder (L); lane 1, CVD30; lane 2, CVD30Δ*cfaB*; lane 3, CVD30Δ*lngA*; lane 4, CVD30Δ*cfaB*Δ*lngA*; lane 5, CVD30Δ*cfaB*(pGA2-CFA/I); lane 6, CVD30Δ*cfaB*Δ*lngA*(pGA2-CFA/I); lane 7, H10407; lane 8, H10407Kan; and lane 9, E9034A.

ETEC clinical isolates were studied to understand CFA/I and CS21 expression in different growth conditions. ETEC H10407 is a well-studied clinical isolate from the 1970s that has been shown to cause severe disease in volunteers [47]. ETEC CVD30 is a recent isolate identified in the GEMS study [10,55]. Both strains contain the genes encoding CFA/I while CVD30 harbors genes encoding CS21 as well. Both H10407 and CVD30 harbor genes encoding STh. ETEC E9034A is the most frequently studied CS21-expressing strain and also encodes STh. The literature supports differing levels of CF expression depending on growth media [23,46]. Using western blot analysis, expression of CfaB, the major subunit of CFA/I, was observed at 15kDa in whole cell lysates of both CVD30 and H10407, but not E9034A, when grown on CFA agar and in Terrific broth (Figs 2 and S1). Both strains expressed higher levels of CFA/I when grown on CFA agar compared to growth in Terrific broth, supporting the use of CFA agar for optimal expression of CFA/I [46]. In both growth conditions, CVD30 expressed more CFA/I than H10407. We noted that CfaB in the purified CFA/I positive control migrated faster than CfaB from the whole cell lysate. We determined that there is a component in the whole cell lysate that lessens migration, since a band is observed similar to that of the whole cell lysate when purified CFA/I is mixed with a whole cell lysate (S1 Fig). LngA, the major subunit of CS21, was observed at 19kDa in whole cell lysates of CVD30 and E9034A, but not H10407, grown on CFA agar and in Terrific broth (Figs 2 and S1). LngA expression by CVD30 was produced at similar levels following growth on each media. This is in contrast to E9034A, which expressed more LngA following growth in Terrific broth than when grown on CFA agar, as previously reported [23]. The purified LngA peptide is tagged with a 6X His tag resulting in a band that runs higher than the whole cell lysates at 21kDa (S1 Fig). These data demonstrate growth condition- and strain-specific CF expression and confirm that ETEC strains can express CfaB and LngA simultaneously in both growth conditions tested.

A series of mutant strains derived from CVD30 were engineered with deletions in *cfaB*, *lngA*, or both *cfaB* and *lngA* genes. The CFA agar and Terrific broth growth conditions were used to confirm the lack of subunit expression in all derivative mutant strains by western blot (Fig 2). CFA/I expression was restored in mutant strains following complementation with pGA2-CFA/I which drives high level expression of CFA/I (Fig 2).

### Simultaneous surface expression of CFA/I and CS21 in wildtype and mutant derivative strains

**Fig 3:**
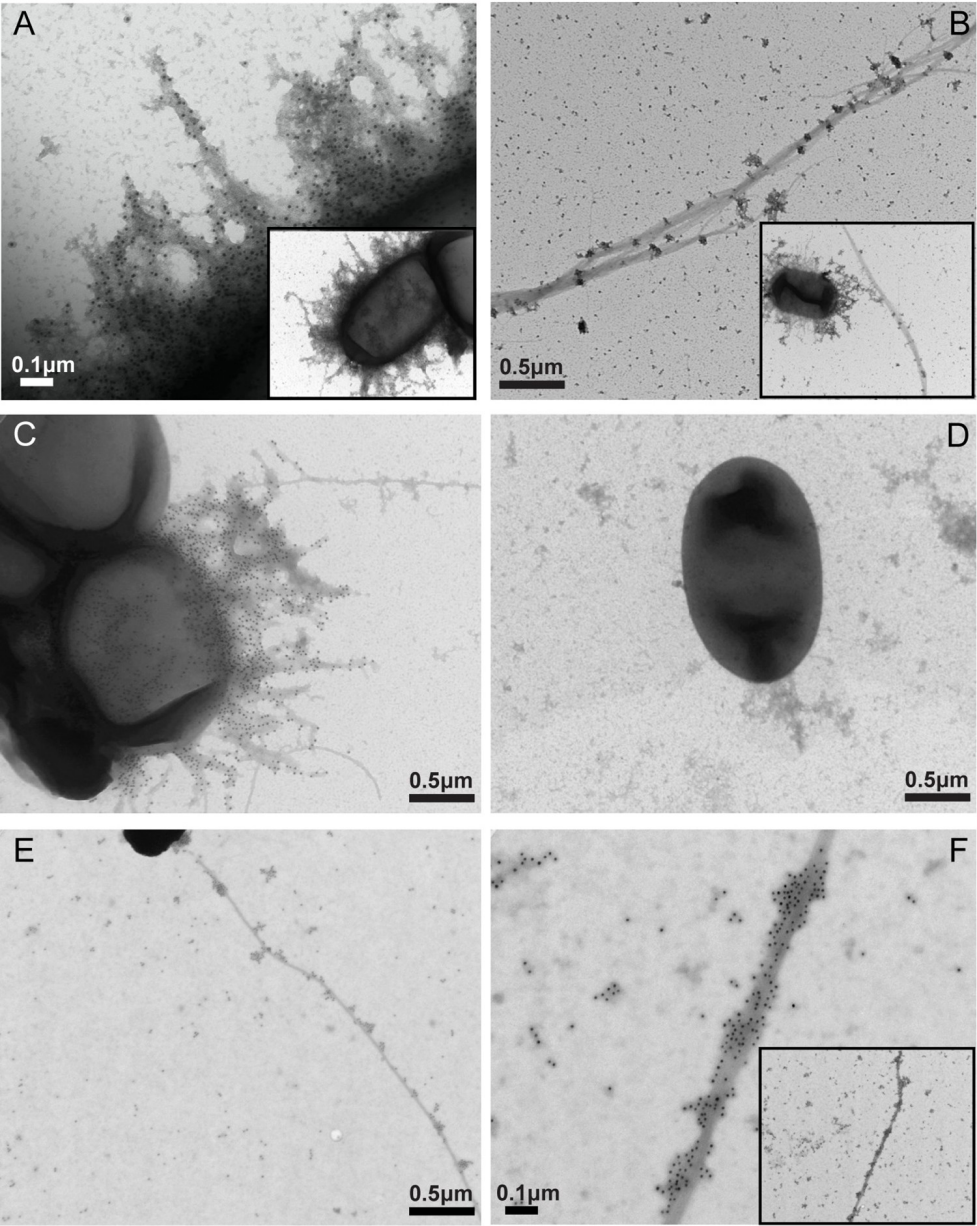
Expression of CFA/I and CS21 on wildtype ETEC strains using immunogold electron microscopy. Bacteria and CFs were visualized by TEM following immunogold staining with CF-specific antibodies. Wildtype strain CVD30 (A, B) was stained with anti-CFA/I antibodies (A) or anti-CS21 antibodies (B). Wildtype strain H10407 (C) and CFA/I-deficient strain H10407Kan (D) were stained with anti-CFA/I antibodies. Wildtype strain E9034A was stained with anti-CS21 antibodies (E, F). All strains were grown on CFA agar. Scale bar size indicated in μM. The insets in panels A and B show the whole bacterium producing the CF of interest covered with gold particles. The inset in panel F shows a lower magnification of the CS21 pili expressed by E9034A covered with gold particles.

**Fig 4:**
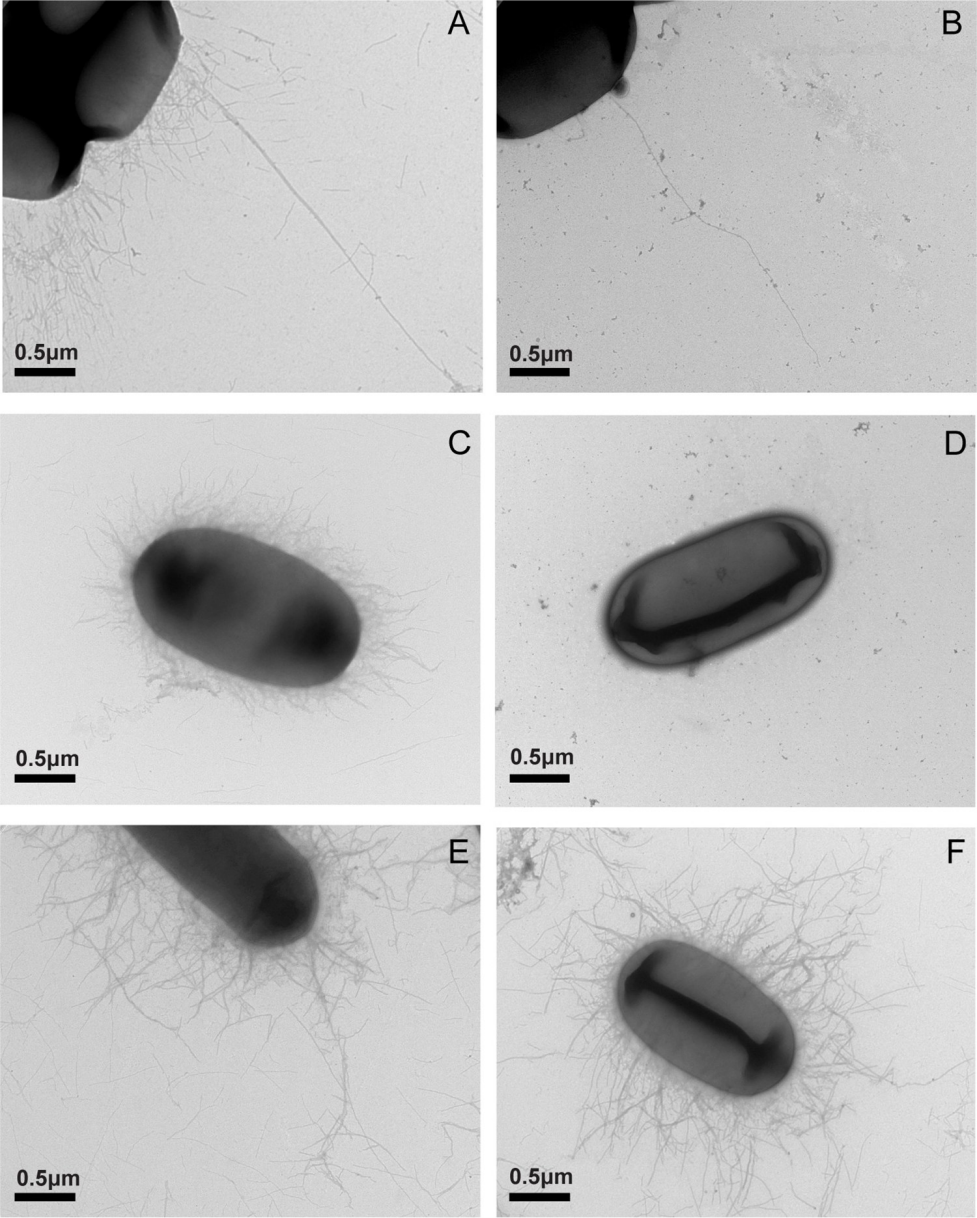
CFA/I and CS21 expression in wildtype CVD30 and derivative mutant strains using negative staining. ETEC strains grown on CFA agar were stained with uranyl acetate and visualized using TEM. Strains are as follows: Wildtype strain CVD30 (A); CVD30Δ*cfaB* (B); CVD30Δ*lngA* (C); CVD30Δ*cfaB*Δ*lngA* (D); CVD30Δ*cfaB*(pGA2-CFA/I) (E); and CVD30Δ*cfaB*Δ*lngA*(pGA2-CFA/I) (F). Scale bar size indicated in μM.

Transmission electron microscopy (TEM) was used to visualize surface expression of CFA/I and CS21 using negative staining and immunogold labeling. Strains were grown on CFA agar, and CF-specific expression was detected using anti-CFA/I and anti-CS21 antibodies. In agreement with western blot findings, both CFA/I and CS21 fimbriae were observed on CVD30, while only CFA/I was observed on H10407 and only CS21 was observed on E9034A (Fig 3). As reported previously, CS21 was observed to be a long bundle-forming pilus, which was in contrast to the peritrichous hair-like projections of CFA/I [27,57]. CFA/I surface expression was not observed in the CFA/I-deficient mutant strains H10407Kan, CVD30*ΔcfaB*, and CVD30*ΔcfaBΔlngA* (Figs 3 and 4). CS21 pili were not observed on the CVD30*ΔlngA* and CVD30*ΔcfaBΔlngA* strains (Fig 4). In addition, CFA/I expression was restored in the complemented mutant strains CVD30*ΔcfaB*(pGA2-CFA/I) and CVD30*ΔcfaBΔlngA*(pGA2-CFA/I), visualized as high-level ectopic expression of CFA/I (Fig 4). These data provide further support for the simultaneous surface expression of CFA/I and CS21 in CVD30 grown on CFA agar.

Agglutination studies were also performed to study the surface expression of specific CFs. Strains were grown on CFA agar, resuspended in PBS, and mixed in equal volumes with anti-CFA/I or anti-CS21 antibodies. H10407 agglutinated with anti-CFA/I, as shown previously [57], while CVD30 agglutinated with anti-CFA/I and anti-CS21 antibodies (Table 3). E9034A agglutinated with anti-CS21 antibodies, as shown previously [27]. In agreement with the western blot studies, CVD30 agglutinated with anti-CFA/I antibodies at an increased level compared to H10407. Agglutination with anti-CFA/I was not observed with the CVD30*ΔcfaB* or the CVD30*ΔcfaBΔlngA* strains, further confirming the loss of CFA/I. Agglutination with anti-CFA/I was restored and resulted in agglutination at a higher level with the complemented derivatives of those mutant strains (Table 3). Agglutination with anti-CS21 was not observed with the CVD30*ΔlngA* and CVD30*ΔcfaBΔlngA* strains, further confirming the loss of CS21 (Table 3). These results support data from western blot and electron microscopy studies and establish the differential expression of CFA/I and CS21 by the wildtype and mutant derivative strains.

**Table 3:** Slide agglutination to detect CF surface expression.

Strain	Agglutination with anti-CFA/I	Agglutination with anti-CS21
H10407	+	−
CVD30	++	++
CVD30Δ*cfaB*	−	+
CVD30Δ*lngA*	++	−
CVD30Δ*cfaB*Δ*lngA*	−	−
CVD30Δ*cfaB*(pGA2-CFA/I)	+++	+
CVD30Δ*cfaB*Δ*lngA*(pGA2-CFA/I)	+++	−
E9034A	−	+

- No agglutination

+ Low level of agglutination with small clumps of bacteria

++ Moderate level of agglutination with mid-large clumps of bacteria

+++ Highest level of agglutination with large clumps of bacteria

### Role of CFA/I in adherence to human enteroid monolayers by ETEC expressing CFA/I and CS21

**Fig 5:**
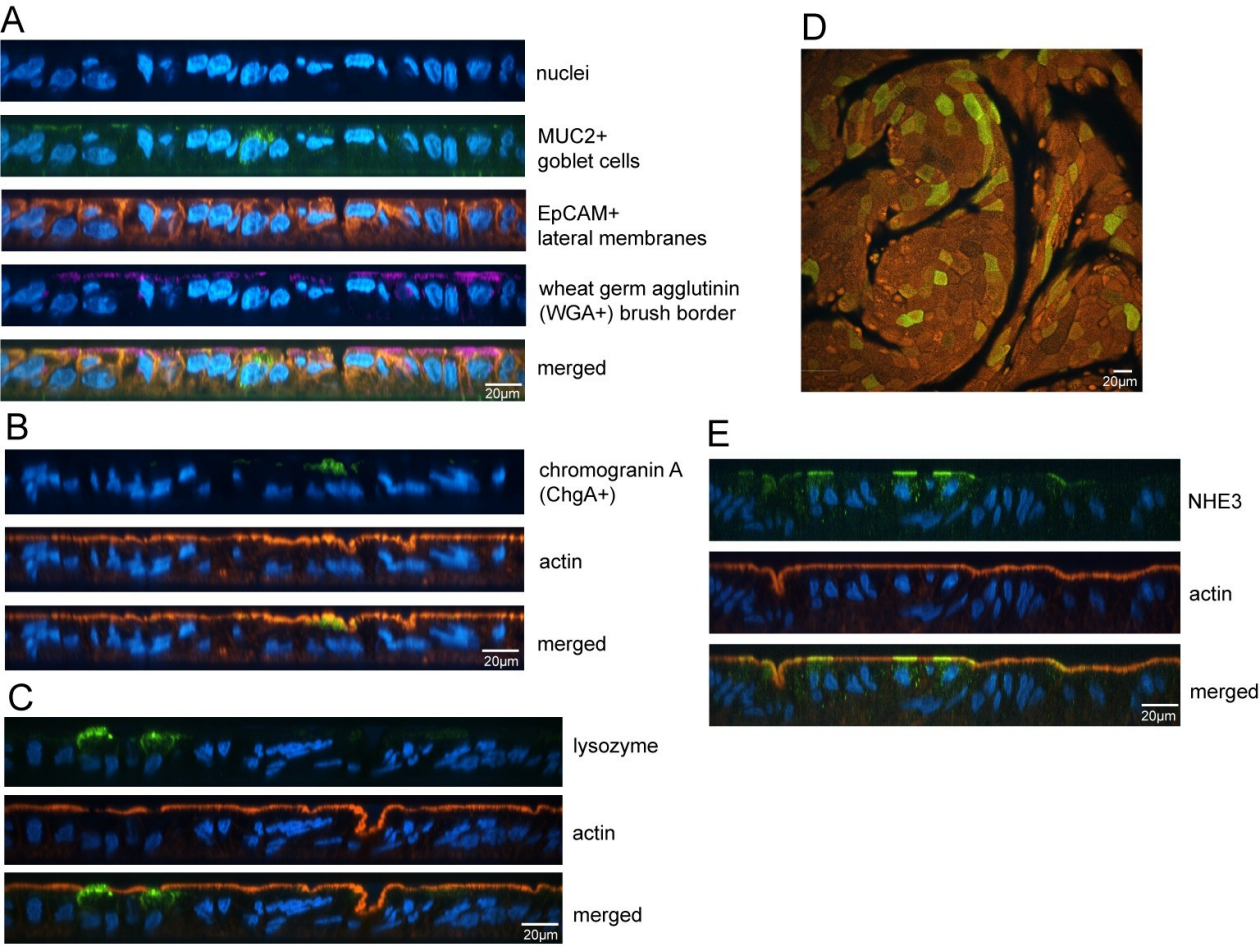
Human ileal enteroids include specialized intestinal cell subtypes. Differentiated enteroid monolayers (35I) were fixed and stained with Hoescht for nuclei in all panels. Monolayers were stained with antibodies against mucin (MUC2) for goblet cells, epithelial cell adhesion molecule (EpCAM) for lateral membranes, and wheat germ agglutinin (WGA) for brush border (A). Monolayers were stained with antibody against chromogranin A (ChgA) for enteroendocrine cells (B). Monolayers were stained with antibodies against lysozyme for Paneth cells (C). Monolayers were stained with antibodies against the NHE3 ion transporter (D, E). Confocal microscopy was used to visualize XY and XZ projections.

**Fig 6:**
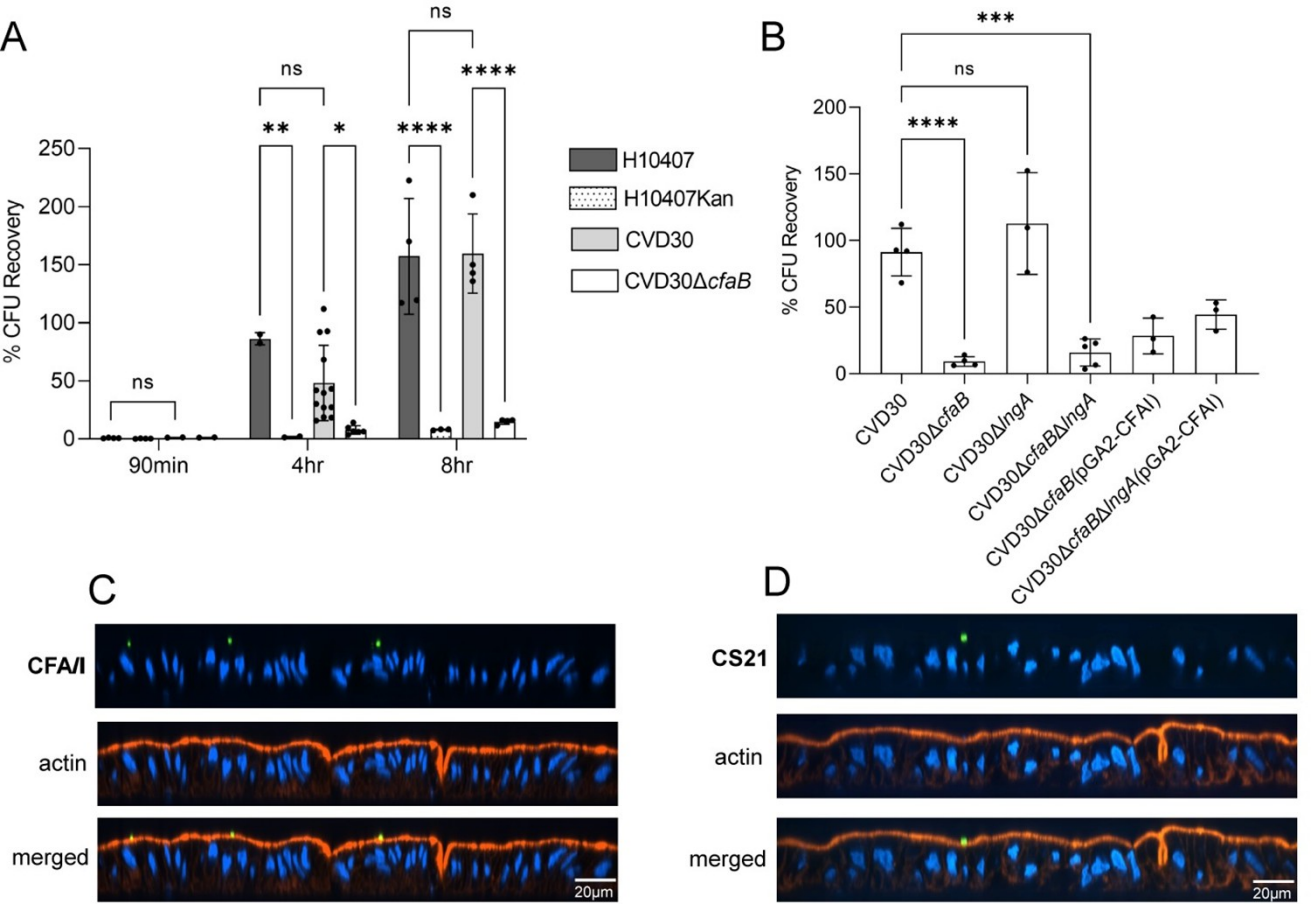
CFA/I mediates adherence by CFA/I-CS21 ETEC to human ileal enteroid monolayers. Differentiated ileal enteroid monolayers (35I) were infected with wildtype ETEC strains or their respective CFA/I-deficient derivative strains for 90 min, 4 hrs, and 8 hrs (A) or infected with wildtype CVD30 or the respective CFA/I- and CS21-deficient strains for 4 hrs (B). Monolayers were washed and lysed to quantify adherent bacteria expressed as % of initial inoculum (CFU recovery). Data presented are pooled from nine independent experiments (A) or two independent experiments (B). Each dot represents data collected from an individual monolayer. Results were confirmed in two additional ileal lines (46I, 34I) and one jejunal line (J2). Error bars indicate standard deviations from the means. The asterisks above the bars indicate statistically significant differences determined using two-way ANOVA (A) or one-way ANOVA (B) with Bonferroni’s multiple-comparison test. *, p<0.05; **, p<0.01; ***, p<0.001; ****, p<0.0001. Monolayers were infected with wildtype CVD30 for 4 hrs, stained with anti-CFA/I (C) or anti-CS21 antibodies (D), and visualized by confocal microscopy (XZ projections).

A multitude of epidemiological, laboratory and clinical data demonstrate that CFA/I is important for ETEC pathogenesis and disease [9,19]. However, data supporting a direct role for CS21 in strains with multiple CFs is unclear. While epidemiological data suggests that CS21-only expressing ETEC are not significantly associated with diarrheal disease [10], previous studies have demonstrated that CS21 is important for adherence to transformed intestinal cell monolayers [23–26]. The high frequency (78%) of CS21 encoded by CFA/I-expressing strains in GEMS leads to questions about the combined role of these two CFs. The contribution of CFA/I and/or CS21 to adherence was assessed using the human enteroid model. Human ileal enteroid monolayers were differentiated to include specialized intestinal cell subtypes, which were visualized by confocal microscopy. Glycosylation epitopes on the brush border and on lateral membranes were visualized by staining for wheat germ agglutinin (WGA) and epithelial cell adhesion molecule (EpCAM), respectively (Fig 5). Mucus-expressing goblet cells (MUC2), chromogranin A-expressing enteroendocrine cells (ChgA), lysozyme-producing Paneth cells, and cells expressing the ion transporter NHE3 were observed (Fig 5). The wildtype clinical strains H10407 and CVD30 as well as the CVD30 derivative mutant strains were grown on CFA agar for simultaneous expression of CFA/I and CS21 (Figs 2–4) and adherence was evaluated over time following apical infection (Fig 6). CFA/I-expressing wildtype strains CVD30 and H10407 demonstrated a time-dependent increase in adherence and, at 4 and 8 hrs post infection, adhered at significantly higher levels than their respective CFA/I-deficient mutant strains (Fig 6). No significant difference in adherence was observed between H10407 and CVD30 to the enteroid monolayer at each timepoint (Fig 6).

Using these established parameters, the roles of CS21 with CFA/I in adherence were investigated using wildtype CVD30 and its derivative mutant strains. At 4 hrs post infection, CVD30*ΔcfaB* adhered significantly less than the wildtype strain while CVD30*ΔlngA* adhered at similar levels. The double mutant CVD30*ΔcfaBΔlngA* also adhered significantly less than the wildtype CVD30 strain which was similar to the decreased adherence of CVD30*ΔcfaB*, supporting the primary role of CFA/I in the adherence of CVD30 to the human enteroid. Complementation of CVD30*ΔcfaB* and CVD30*ΔcfaBΔlngA* with pGA2-CFA/I resulted in restored adherence that was increased but not significant compared to mutant strains while not reaching WT levels (Fig 6). It was noted that the complemented strains grew more slowly, likely due to the metabolic burden of the high-level CFA/I expression (Fig 4). The observed increased CFA/I expression by the complemented mutants (Table 3, Fig 4) may result in hyper-agglutination of the bacteria and account for reduced attachment to the monolayer. The complemented mutants exhibited slightly lower growth in DMEM compared to the wildtype CVD30 strain (S2 Fig). Adherence to enteroid monolayers by CVD30 was visualized by confocal microscopy, and CFA/I and CS21 expression was observed during infection at 4 hrs (Fig 6). The adherence data were confirmed using two additional ileal and jejunal enteroid lines derived from other individuals, supporting the power of the enteroid model to confirm the role of CFs in attachment in genetically diverse individuals (S3 Fig).

### The role of anti-CFA/I and anti-CS21 antibodies in adherence inhibition of CFA/I-CS21 ETEC to human enteroid monolayers

**Fig 7:**
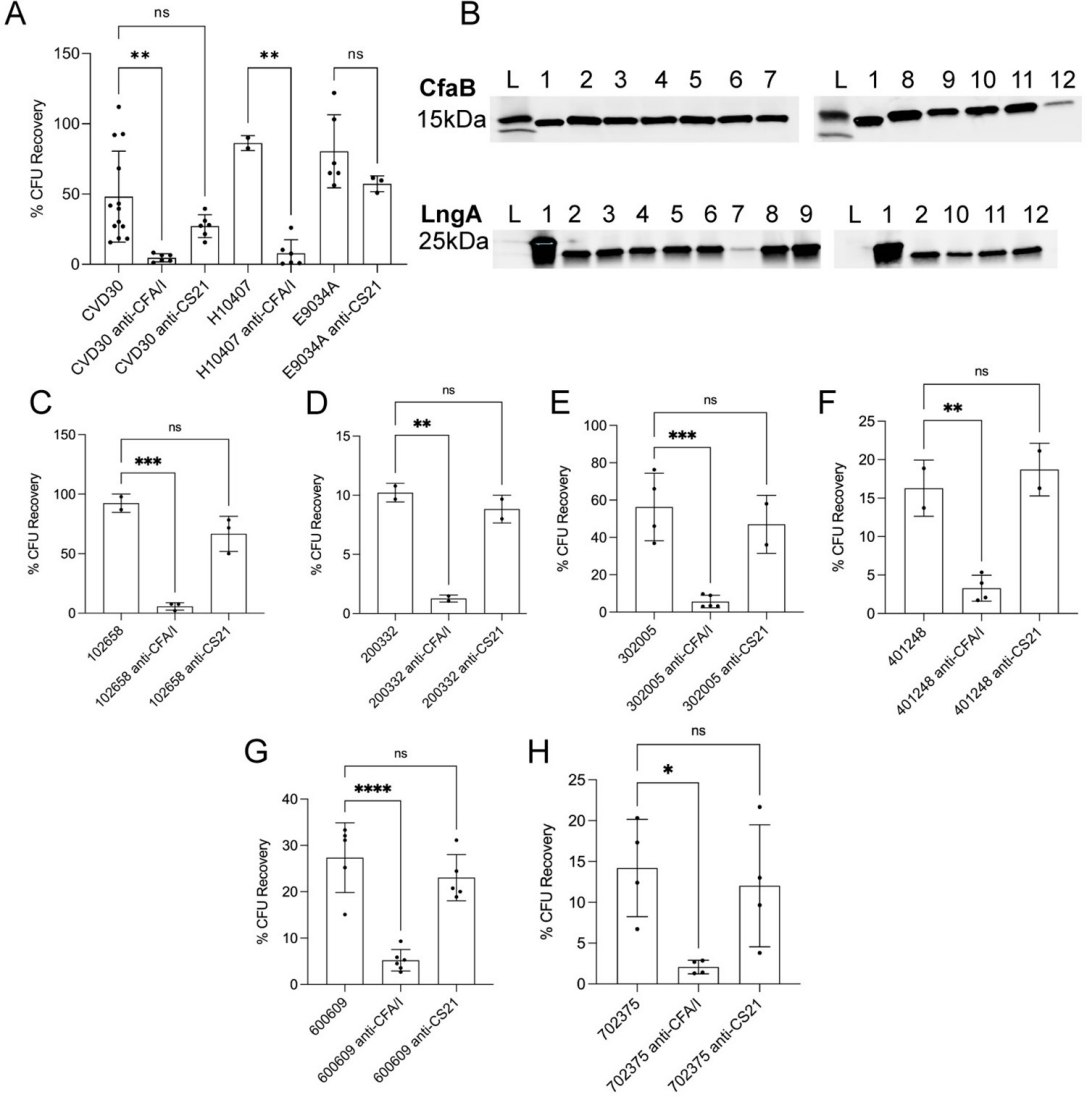
Anti-CFA/I antibodies but not anti-CS21 antibodies inhibit adherence to human ileal enteroid monolayers by ETEC strains expressing CFA/I and CS21. Differentiated ileal enteroid monolayers (35I) were infected with wildtype ETEC strains CVD30, H10407, or E9034A (A) or six different strains (102658, 200332, 302005, 401248, 600609, and 702375) from other geographical regions (C–H) for 4 hrs with or without pre-incubation with anti-CFA/I or anti-CS21 antibodies. Monolayers were washed and lysed to quantify adherent bacteria expressed as % of initial inoculum (CFU recovery). Data presented are pooled from ten independent experiments (A) or two independent experiments (C–H). Each dot represents data collected from an individual monolayer. Results in A were confirmed in a jejunal enteroid line (J2). Error bars indicate standard deviations from the means. The asterisks above the bars indicate statistically significant differences determined using one-way ANOVA with Bonferroni’s multiple-comparison test. *, p<0.05; **, p<0.01; ***, p<0.001; ****, p<0.0001. (B) ETEC strains were grown on CFA agar, and whole cell bacterial lysates were probed with anti-CFA/I (top) or anti-CS21 (bottom) antibodies. Ladder (L); lane 1, purified protein (CFA/I 500ng (top), LngA 300ng (bottom)); lane 2, CVD30; lane 3, 100386; lane 4, 102658; lane 5, 200332; lane 6, 204033; lane 7, 300202; lane 8, 302005; lane 9, 401248; lane 10, 600609; lane 11, 702213; and lane 12, 702375. Purified CFA/I results in a CfaB band that migrates faster than that in whole cell lysates. Purified LngA is tagged by a 6X His tag (21kDa) while LngA in whole cell lysates result in a band at 19kDa.

The demonstration that anti-CF responses decreased diarrheal disease in human challenge studies has supported vaccine development efforts that target CFs [19,38]. We quantified the adherence-blocking ability of CFA/I- and CS21-specific antibodies against wildtype strain CVD30 expressing both CFA/I and CS21. Strains grown on CFA agar were incubated with either anti-CFA/I or anti-CS21 antibody and used to infect human enteroid monolayers for 4 hrs. Anti-CFA/I antibody inhibited adherence of CVD30 by 88% compared to the wildtype strain alone (Fig 7). In contrast, anti-CS21 antibody did not significantly decrease adherence by CVD30 (Fig 7). The importance of the CFA/I-blocking antibody was further demonstrated with wildtype strain H10407, in which adherence was decreased by 79% (Fig 7). Interestingly, E9034A adherence was not inhibited in the presence of anti-CS21 antibody, which contrasts with CS21 adherence studies performed in cell culture models [23–26] (Fig 7). This may be due to the expression of the major CF CS3 that E9034A also contains [27,48]. These data were confirmed using a jejunal line derived from another individual (S4 Fig). To determine if the level of CS21 expression impacts the ability of anti-CS21 antibodies to inhibit adherence, wildtype ETEC strains E9034A and CVD30 were grown in Terrific broth to increase CS21 expression (Fig 2), pre-incubated with anti-CS21 antibodies, and used to infect human enteroid monolayers for 4 hrs. Despite the increased CS21 expression by these strains, the anti-CS21 antibody did not significantly decrease adherence by E9034A or CVD30 (S5 Fig). Control normal rabbit serum did not inhibit adherence by CVD30 (S6 Fig).

CFA/I-CS21 expressing ETEC strains were isolated from children with MSD from all geographic sites surveyed in GEMS [1]. A set of geographically diverse isolates, two from Asia and four from different countries in Africa, were assessed to further understand the global significance of CFA/I and CS21 on adherence in addition to the studies with CVD30 isolated from India. All isolates were grown on CFA agar and expression of CFA/I and CS21 was demonstrated by western blot (Fig 7). Global ETEC isolates were pre-incubated with anti-CFA/I or anti-CS21 antibodies and used to infect human enteroid monolayers for 4 hrs. Incubation with anti-CFA/I antibody caused a significant decrease in adherence by all six global isolates 102568, 200332, 302005, 401278, 600609, and 702375 by 94%, 89%, 90%, 88%, 78%, and 85%, respectively, while incubation with anti-CS21 antibody did not affect adherence (Fig 7), thus underscoring the impact of targeting CFA/I with strains with multiple CFs across diverse geographical regions. To assess adherence by CFA/I-CS21 ETEC isolated from South America, an ETEC isolate 10002a, from Chile, was pre-incubated with anti-CFA/I or anti-CS21 antibodies and used to infect human enteroid monolayers for 4 hours. Similar to the other global isolates, incubation with anti-CFA/I antibodies caused a significant decrease in adherence by 78% by ETEC 10002a while incubation with anti-CS21 antibodies did not affect adherence (S7 Fig).

### Investigation of the role of CFA/I and CS21 in ST delivery and polarized cGMP secretion in human enteroid monolayers

Multiple studies using transformed cell lines including T84 or Caco-2 have demonstrated cGMP production following treatment with purified ST or infection with ETEC [7,11,35,36,58]. Recent studies using human enteroids demonstrated differential localization and secretion of cGMP between enteroids and cell lines [35]. Enteroids retained less intracellular cGMP than transformed cell lines and secreted higher levels in the basolateral than apical compartments. This marks an interesting difference from data previously generated in transformed cell lines and supports the physiological implications of the enteroid model.

**Fig 8:**
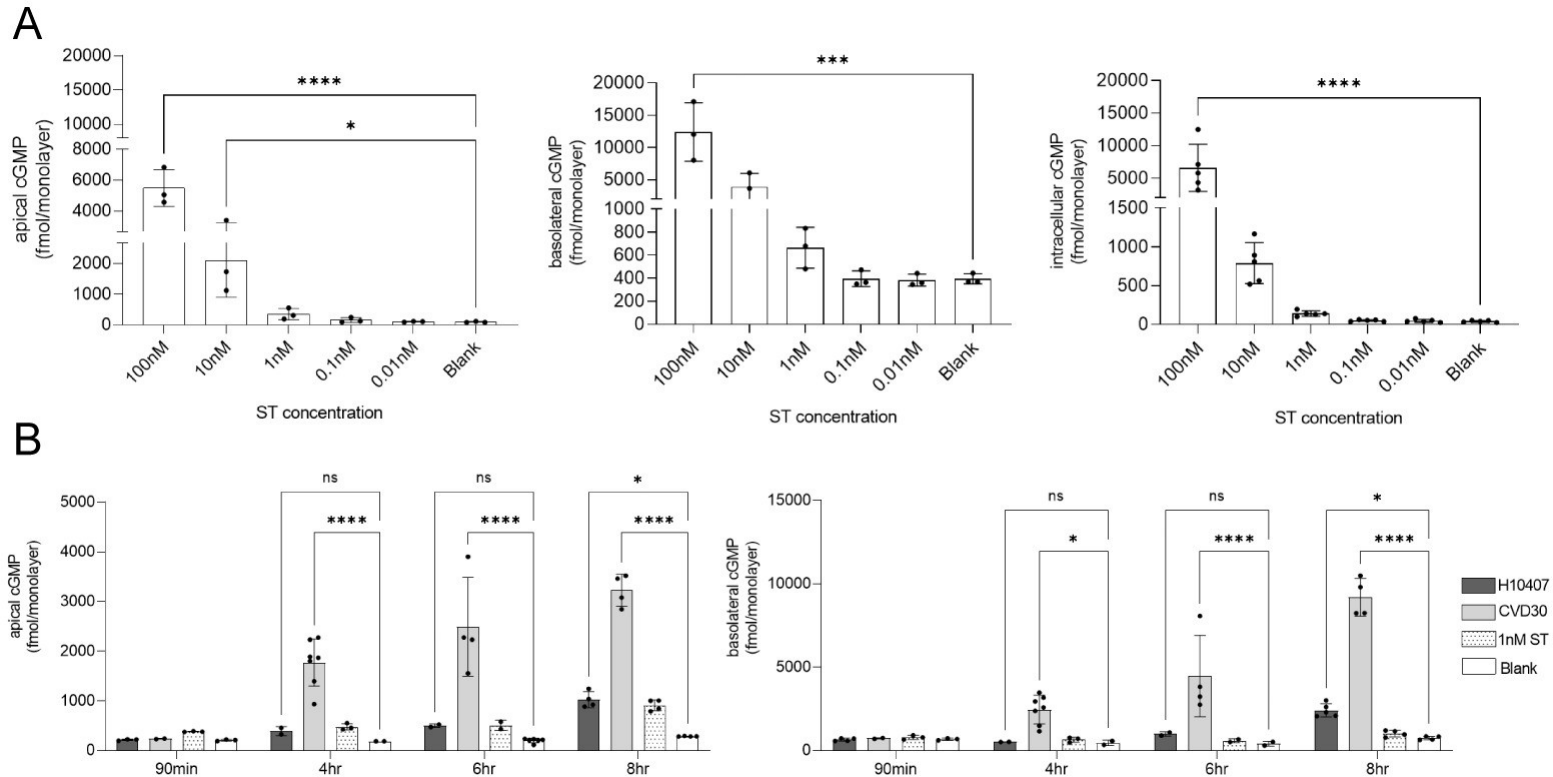
The polarized production of cGMP in human ileal enteroid monolayers following purified ST toxin exposure or infection by wildtype ETEC clinical isolates. Differentiated ileal enteroid monolayers (35I) were exposed to different concentrations of purified ST toxin for 8 hrs (A) or infected with wildtype strains H10407 or CVD30 from 90 min to 8 hrs (B). Apical and basolateral supernatants and intracellular lysates were collected for cGMP ELISA. Data presented are pooled from three independent experiments (A) or eight independent experiments (B). Each dot represents data collected from an individual monolayer. Error bars indicate standard deviations from the means. The asterisks above the bars indicate statistically significant differences determined using one-way ANOVA with Tukey’s multiple-comparison test (A) or two-way ANOVA with Bonferroni’s multiple-comparison test (B). *, p<0.05; ***, p<0.001; ****, p<0.0001.

We first tested a dilution series of purified ST incubated with ileal enteroid monolayers and demonstrated dose-dependent cGMP production in the intracellular, apical, and basolateral compartments (Fig 8). At 8 hrs post incubation with ST, the cGMP levels were highest in the basolateral compartment as compared to the apical and intracellular compartments in accordance with previous toxin studies in the enteroid model [12]. We next demonstrated that infection with CVD30 and H10407 (10^6^ bacteria) induced time-dependent increases in cGMP levels from 90 min to 8 hrs in enteroids with the highest levels of cGMP recorded in basolateral supernatants (Fig 8). Wildtype strain CVD30 induced high levels of cGMP in both compartments as early as 4 hrs. In contrast, CFA/I-only wildtype strain H10407 did not induce significant levels of cGMP until the 8 hr timepoint in both apical and basolateral compartments, matching the delayed basolateral cGMP production reported previously (Fig 8) [35]. H10407 secretes less ST than CVD30, which most likely accounts for decreased levels of cGMP induced between strains (S8 Fig) [55]. These data demonstrate that the enteroid model can detect strain-specific differences in ST delivery.

**Fig 9:**
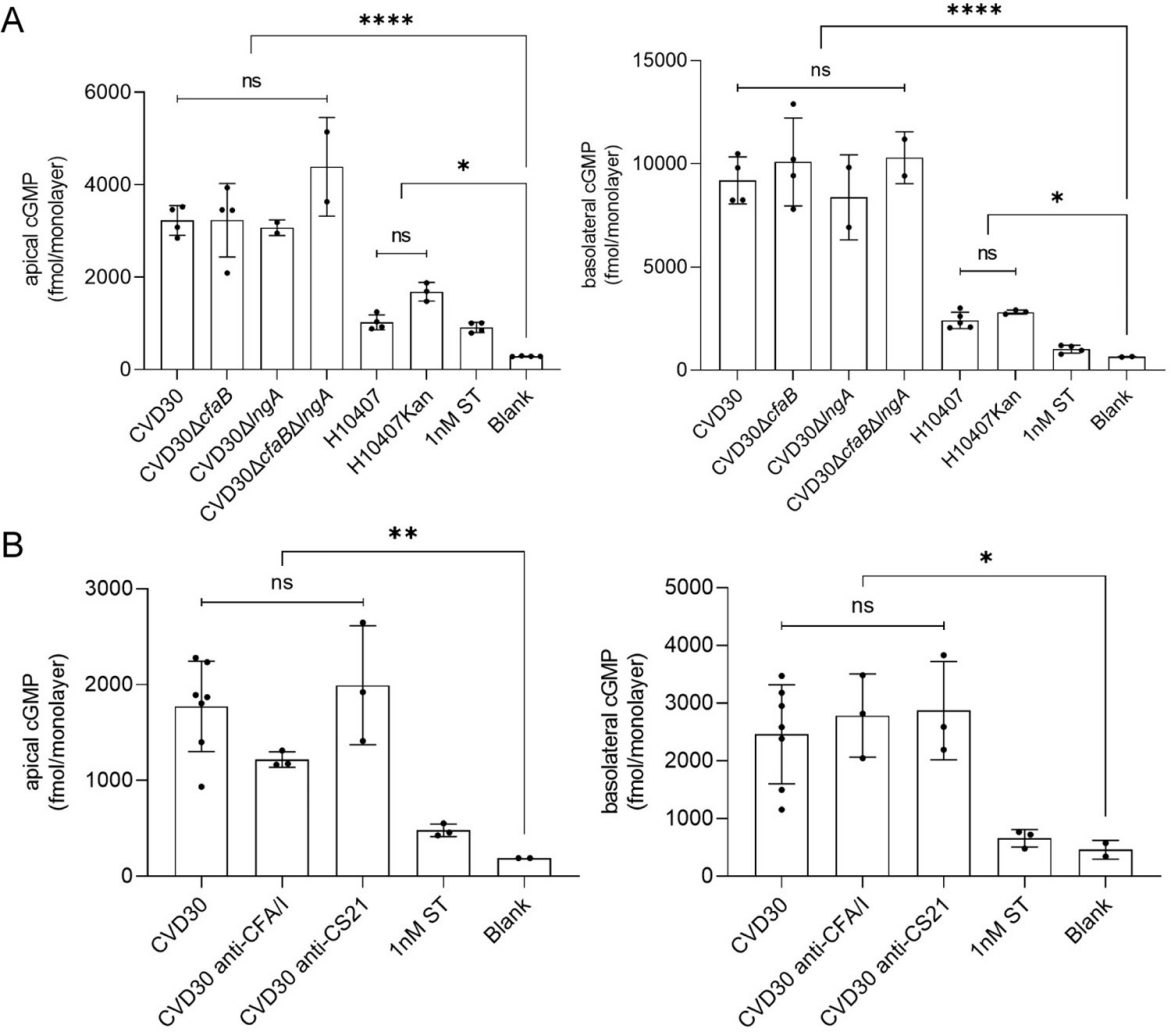
The polarized production of cGMP in human ileal enteroid monolayers following infection by CVD30 and CF-deficient mutant strains. Differentiated ileal enteroid monolayers (35I) were infected with wildtype strain CVD30 or CFA/I- and CS21-deficient strains for 8hrs (A) or infected for 4 hrs with wildtype strain CVD30 with or without pre-incubation with CFA/I- or CS21- antibodies (B). Apical and basolateral supernatants were collected for cGMP ELISA. Data presented are pooled from two independent experiments (A) or four independent experiments (B). Each dot represents data collected from an individual monolayer. Toxin delivery results were confirmed in one jejunal enteroid line. Error bars indicate standard deviations from the means. The asterisks above the bars indicate statistically significant differences determined using one-way ANOVA with Bonferroni’s multiple-comparison test. *, p<0.05; **, p<0.01; ****, p<0.0001.

A few reports describe the effect of CFs, as well as other adhesins, and virulence factors on ST delivery [35,36]. However, the role of multiple CFs, specifically CFA/I and CS21, on ST delivery has not been examined. Enteroid monolayers were apically infected with wildtype or CF-deficient bacteria for 8 hrs and secreted cGMP production was measured in the apical and basolateral supernatants. CVD30 induced high levels of cGMP with greater levels of cGMP measured in basolateral versus apical supernatants (Fig 9). However, all derivative mutant strains CVD30*ΔcfaB*, CVD30*ΔlngA*, and CVD30*ΔcfaBΔlngA* induced similar cGMP levels as the wildtype strain CVD30, suggesting that CFA/I and CS21 do not play a role in ST delivery in this model (Fig 9). Studies with H10407 demonstrated similar results with comparable induction of cGMP by wildtype and CFA/I-deficient mutant strain H10407Kan (Fig 9). Furthermore, neither of the CF-specific antibodies interfered with cGMP production in enteroids infected with wildtype CVD30 after 4 hrs (Fig 9). These data were confirmed using a jejunal enteroid line (S9 Fig). Given that adherence and toxin delivery are responsible for the clinical symptoms of ETEC diarrheal disease, the lack of observed CF-dependent toxin delivery suggests that the human enteroid model has limitations that impact intoxication due to CF-dependent attachment. Some enteroid studies suggest that incorporating forces, such as flow and stretch, can influence enteroid differentiation and cGMP production, which may affect the response to ST delivery by ETEC [59,60]. This is in agreement with previous reports that concluded that ST delivery and downstream cGMP production by H10407 did not entirely correlate with adherence alone; other virulence factors unrelated to adherence, including EatA, were shown to induce decreased cGMP [35]. An additional limitation of this study’s enteroid model is the exclusive aerobic conditions. Gene expression studies in ETEC isolated from human stool samples in a controlled human infection model determined that oxygen directly impacted regulation of classical virulence factor expression [61].

### The effects of CFA/I-CS21 ETEC infection on human intestinal enteroid physiology

**Fig 10:**
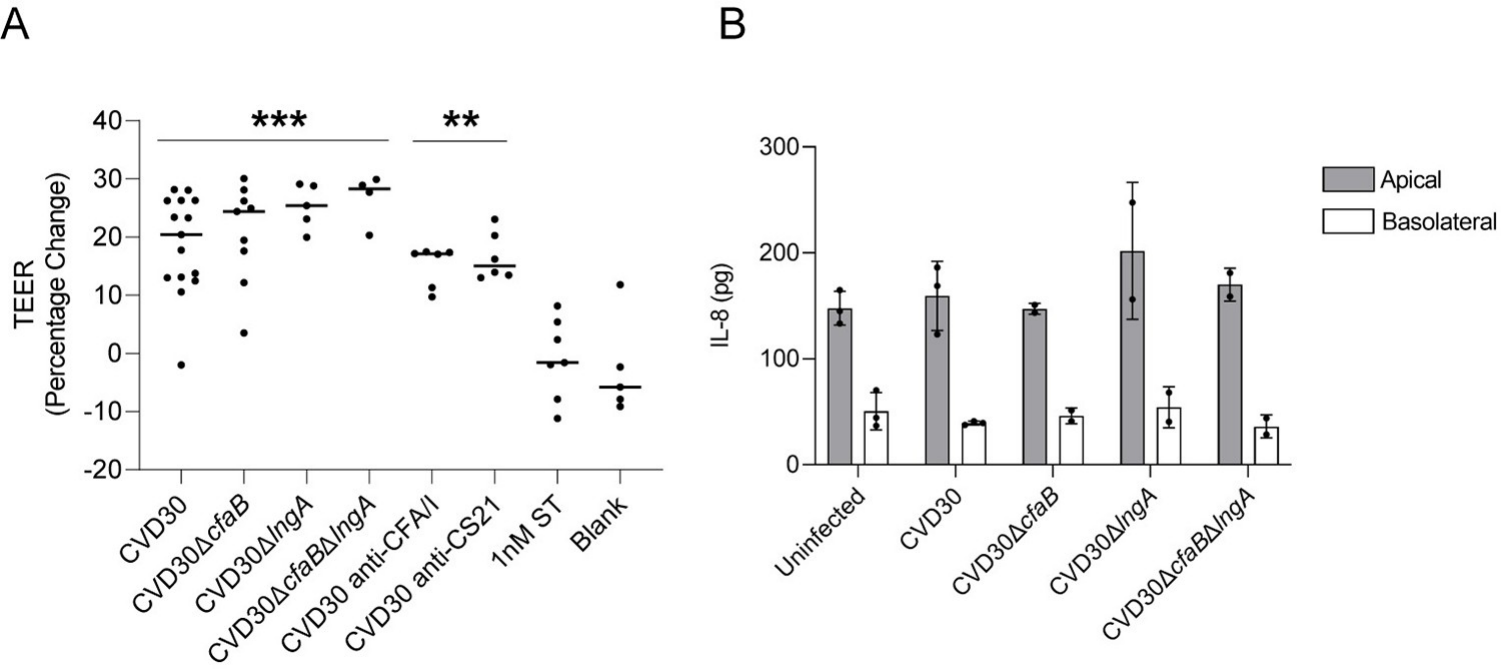
The effect of CFA/I-CS21 ETEC infection on barrier integrity and cytokine induction in ileal enteroid responses. TEER values of differentiated ileal enteroid monolayers (35I) were recorded prior to and after 4 hr infection (A). The change in TEER over time is expressed as TEER (percentage change) and data presented are pooled from six independent experiments. The asterisks above the dots indicate statistically significant differences determined using one-way ANOVA with Bonferroni’s multiple-comparison test, compared to the TEER of the uninfected enteroid monolayers (Blank). **, p<0.01; ***, p<0.001. IL-8 secretion (pg) was measured following 8 hr infection of enteroid monolayers (35I) by wildtype CVD30 and its derivative CF-deficient mutant strains, and data presented are from one independent experiment (B). Each dot represents data collected from an individual monolayer. Statistically significant differences were calculated using one-way ANOVA with Bonferroni’s multiple-comparison test.

ETEC is an adherent, noninvasive enteric pathogen, and its effect on the intestinal barrier function during infection is not completely understood. Previous studies using the T84 colonic transformed cell line suggest that ETEC toxins LT and ST alone as well as LT-expressing ETEC negatively affect the transepithelial electrical resistance (TEER) [62,63]. However, recent work using enteroids reported no cytotoxicity or changes in the TEER following exposure to ST toxin or infection by wildtype H10407 and derivative strains [11,35]. To further investigate enteroid barrier integrity following infection with the recent clinical isolate CVD30 and its CF-deficient mutant strains, TEER values of ileal enteroid monolayers were measured prior to and 4 hrs post infection. The TEER remained unchanged following ST toxin exposure and similar to uninfected monolayers; however, the TEER was significantly increased in all monolayers infected with ETEC, regardless of CF expression (Fig 10). There was no significant difference between the TEER levels of enteroid monolayers infected with bacteria alone or bacteria pre-incubated with anti-CF antibodies (Fig 10). Also, the TEER levels following 4 hr infection by ETEC strains in an additional ileal enteroid line was consistently increased compared to uninfected monolayers (S10 Fig).

There are few reports on the host innate immune response to ETEC infection. Early studies in pigs suggested that ETEC infection resulted in a pro-inflammatory immune response with high levels of TLR4 signaling and an overproduction of cytokines, including IL-6 and IL-8 [64,65]. Studies in T84 cells reported low levels of IL-8 in response to ST toxin at later timepoints (18 hrs) [11]. In contrast, other studies determined that ETEC directly inhibits the innate immune response by blocking NF-κB signaling, and that overnight infection with ETEC did not affect IL-8 secretion in ileal enteroids [52,66]. To investigate the role of CFA/I and CS21 on IL-8 production using the enteroid model, monolayers were infected for 8 hrs with wildtype CVD30 and the CF-deficient mutant strains, and cytokine levels in supernatants were measured by ELISA. There was no difference in IL-8 production between uninfected monolayers and those infected with any bacterial strain, regardless of CF expression (Fig 10).

## Discussion

This study underscores the importance of the major ETEC CF CFA/I alone, or when co-expressed with CS21, in ETEC pathogenesis and as an essential target for adherence-blocking vaccine strategies, as no change in function is found when CS21 was present or not. Given the high prevalence of CS21 in ETEC isolates expressing CFA/I in the GEMS study (78%), this study investigated the combined effects of two CFs on key features of ETEC pathogenesis including adherence and toxin delivery [10]. The use of the enteroid model provided a highly human-relevant system for investigation.

Experiments with CF-specific mutants as well as CF-specific antibodies demonstrated that CFA/I was critical for adherence while CS21 did not play a significant role. The ability of anti-CFA/I antibodies to block adherence supports vaccine strategies that include CFA/I to target ETEC strains with multiple CFs. The GEMS finding that CS21 alone was not significantly associated with MSD in young children supports these data [10]. However, these data are not in agreement with other studies using transformed cell lines where LngA mutants had decreased adherence compared to the wildtype strain and/or anti-CS21 antibodies were shown to block CS21-expressing ETEC attachment [23–26].

The differing results in the role of CS21 may be due to growth conditions. This study used CFA agar as the primary growth condition to investigate adherence and toxin delivery because of the high level of CFA/I and CS21 expression as shown by western blots, electron microscopy, and agglutination. Terrific broth static growth conditions were also used to increase CS21 expression in wildtype strains and test the ability of anti-CS21 antibody to inhibit adherence. However, previous adherence studies have used ETEC strains grown in PPLO broth overnight to demonstrate CS21-mediated attachment to transformed cell lines [25,26].

Another important distinction is that previous studies used models, including porcine epithelial cells (IPEC), HT-29 cells, and neonatal mice, which may all contain different surface glycans than those found on the human intestinal surface and can likely impact CF-mediated adherence [25,26]. While a receptor for specific CS21 has not been identified, previous studies in transformed cell lines and human intestinal tissue suggest that ETEC CFs and pili contain lectin domains that recognize glycoconjugates or glycosphingolipids on the host surface [67–69] and studies of type IVb pili demonstrate that ETEC expressing type IVb pili bind to glycoconjugates on human intestinal biopsies from the small and large intestine [70,71]. Identifying glycans that serve as receptors for pathogens in the enteroid model is currently understudied; however, recent but limited work in enteroids has demonstrated the expression of important receptors for host-pathogen interactions [36,72,73].

It is also important to note that the aforementioned transformed cell models are not differentiated to include specific cell subtypes similar to the enteroid; it is likely that they lack important proteases and other secreted factors that may affect CF-mediated ETEC adherence. Continued progress in the research of differentiated enteroid culture aims to express additional cell types not yet represented, including transit-amplifying cells and stem cells. However, given the observed role of CS21 in transformed cell lines, it is likely that CS21 binds to a glycan expressed by epithelial cells already present in enteroid monolayers.

While our study utilized growth conditions that resulted in co-expression of both CFs, regulation of CF expression in the human intestine is not clear. Detection of anti-CS21 antibodies in ETEC-infected individuals supports its expression in the human host [39]. However, it is unclear if CS21 is co-expressed with other CFs, particularly CFA/I, during human infection. Our studies may support an alternative role for CS21 during ETEC pathogenesis, which may include biofilm formation and twitch mobility as previously demonstrated [23].

Given that this study primarily focused on ETEC strains isolated from countries in Asia and Africa [10], future studies should include strains isolated from the Americas, in which CS21 is highly prevalent in ETEC isolates based on previous epidemiological studies in children under five and travelers [26,74–81]. It is important to consider CS21+ ETEC strains from the Americas given that they may contain different CS21 sequences, particularly in LngA, and have different binding characteristics than those described in this study.

These studies demonstrated that CFA/I-mediated adherence was critical to wildtype strains with one CF (H10407) as well as those with multiple CFs (CVD30 and additional global ETEC isolates). Interestingly, CVD30 consistently expressed more CFA/I than H10407 in all methods tested, despite both strains containing 99.9% identical CFA/I operon sequences, including the upstream promoter sequence, and 99.87% identical regulator CfaD sequences. Regulation of the CFA/I operon in these strains warrants further investigation. Key regulators that affect CF expression, including Rns, Crp, Hns, CpxR, FNR, and IscR, are present in both H10407 and CVD30, and sequences are 100% identical [9,55,61,82]. It is also possible that post-transcriptional regulatory mechanisms or small inhibitory RNA may affect the differential CFA/I expression in these strains, for which there are limited studies in ETEC.

Antibody inhibition experiments were performed using bivalent agglutinating anti-CF antibodies. Many studies have employed this approach to study adherence mediated by other CFs, including CS2 [83], CS3 [83], CS6 [54] and CS21 [23,25]. The ability of agglutinating antibodies to inhibit ETEC disease has also been demonstrated in human clinical trials [37]. However, it is important to note the shortcomings of using agglutinating antibodies given the potential disruption of fimbrial physical properties including aggregation and steric hinderance [84]. Previous studies have used Fab fragments to definitively demonstrate CF component-specific binding inhibition [85,86], and future studies may use Fab fragments specific to CFs of interest to minimize the impact on the fimbriae.

The use of enteroids allowed quantification of ST-dependent production and secretion of cGMP detected in apical and basolateral compartments. As previously reported [11,35], cGMP accumulated at higher levels in the basolateral supernatants and less in apical supernatants. Our experimental conditions resulted in quantification of cGMP following infection with 10^6^ CFU of CVD30 as early as 4 hrs in both compartments that subsequently increased over time. Strain H10407, which has been shown to produce less ST, induced cGMP at 8 hrs post infection in both compartments [55]. These results support the sensitivity of the enteroid model for quantifying ETEC strain-specific differential toxin expression. To note, previous studies utilizing infection with 10^8^ CFU of H10407 reported significant cGMP levels only in basolateral supernatants starting at 6 hrs post infection [35]. Our study demonstrated that H10407 induced higher levels of cGMP in both compartments at 8hrs than reported previously [35]. The observed differences of cGMP levels between these studies may be due to the bacterial growth condition and its effect on ST production and secretion.

Clinical trial data demonstrating that blocking CFs prevents diarrheal disease supports the requirement for attachment-mediated toxin delivery [19,38]. However, CF-mediated toxin delivery in the enteroid model was not evident. The finding that cGMP induction was not decreased following infection with CFA/I- and/or CS21-deficient mutant strains nor following pre-incubation with CF-specific adherence-blocking antibodies reflect a shortcoming of this system. The enteroid monolayer model is static and does not include the intestinal features of flow and stretch. We attempted various washing protocols following bacterial infection that addressed toxin delivery timing, saturation of toxin as a consequence of bacterial inoculum, and plate movement during the experiment, but we could not mimic *in vivo* conditions that would reflect the requirement for bacterial adherence for toxin delivery and disease [87,88]. Similarly, other studies with H10407 failed to demonstrate toxin delivery solely mediated by CFA/I given that other adherence factors, such as EatA and EtpA, were observed to affect toxin delivery to enteroid monolayers [17,35,36] as well as in transformed cell lines [36,89,90]. Intestinal flow has been shown to be important for gut physiology [91] and the use of flow chambers in future studies is expected to reveal CF-dependent ST delivery due to increased differentiation of the enteroid monolayers and increased sensitivity to purified ST toxin [59,60]. Another limitation of the enteroid model in this study is the exclusive aerobic culture conditions. ETEC isolated from stool samples in a controlled human infection model determined that gene expression from these samples more closely matched ETEC grown in anaerobic conditions [61]. These low oxygen conditions directly affected expression of the regulator FNR which then directly affected global virulence gene expression in ETEC, including the CFA/I operon and LT and ST genes [61]. Further advancements to the enteroid model are expected to recapitulate the intestinal environment more appropriately, such as including an anaerobic environment with shear stress consistent with flow and stretch.

In addition to the study of adherence and toxin delivery by ETEC, enteroids provide a model for studying effects on barrier integrity and innate responses following ETEC infection. In this study, we showed that monolayers remained intact and increased in TEER levels during ETEC infection, demonstrating the absence of barrier disruption and suggesting increased expression of proteins important for membrane permeability [92]. This study also demonstrated a muted induction of the innate immune response by ETEC, as shown by the lack of IL-8 production following 8 hr infection. This is in direct contrast to other enteric pathogens including *Shigella* and EAEC that disrupt barrier function during pathogenesis [93–95]. These pathogens cause significant barrier disruption in the enteroid, secretion of IL-8, and high levels of innate cell recruitment, which is in direct contrast to the minimal innate response caused by ETEC. Further studies using the enteroid model are important to better understand the early host response to ETEC.

Vaccine development for ETEC is challenging due to the diversity of virulence factors, especially colonization factors that mediate intestinal adherence. Current strategies demonstrate promise in targeting major CFs as vaccine antigens to block colonization and disease [41,42,45]. In particular, the GEMS study concluded that inclusion of all major CFs in a vaccine would prevent up to 66% of pediatric MSD cases due to ST-only ETEC in LMICs and did not support inclusion of minor CFs with the exception of CS14 [10]. Despite the presence of CS21 in these CFA/I-CS21 strains and previous literature suggesting a role in CS21-mediated attachment [23–26], blocking CFA/I resulted in significantly decreased adherence by ETEC strains expressing CFA/I and CS21. This result was consistent across other global ETEC isolates, emphasizing the universal effectiveness of this strategy. Importantly, these data were determined using four different enteroid lines from the small intestine, three from the ileum and one from the jejunum, with all blood types represented, emphasizing the genetic diversity of the host model used. This study underscores the value of the human enteroid model to study ETEC pathogenesis as well as supports a vaccine strategy that focuses on targeting major CFs, especially CFA/I, to decrease the burden of ETEC-mediated morbidity and mortality across the globe.

## Supporting information

S1 Fig Expression of CFA/I and CS21 by purified CF proteins and wildtype ETEC strains. Purified CFA/I fimbriae were run alone and mixed with whole cell lysate alongside whole cell lysates of wildtype strains CVD30, H10407, and E9034A grown on CFA agar. The blot was probed with anti-CFA/I antibodies (A). Purified CFA/I results in a CfaB band that migrates faster than that in whole cell lysates. Purified LngA peptide was titrated and whole cell lysates of wildtype strains CVD30, E9034A, and H10407 grown in Terrific broth static conditions overnight were run on a western blot probed with anti-CS21 antibodies (B). Purified LngA is tagged by a 6X His tag (21kDa) while LngA in whole cell lysates result in a band at 19kDa.
(TIFF)

S2 Fig Growth curve of complemented CFA/I mutants compared to wildtype CVD30 in DMEM. Growth curve of ETEC strains CVD30 and complemented mutants CVD30Δ*cfaB*(pGA2-CFA/I) and CVD30Δ*cfaB*Δ*lngA*(pGA2-CFA/I) was assessed at 37°C with shaking in DMEM. Growth was monitored every 30 min by measuring OD_600_.
(TIFF)

S3 Fig CFA/I mediates adherence by CFA/I-CS21 ETEC to additional ileal and jejunal human enteroid monolayers. Differentiated jejunal and ileal enteroid monolayers 46I (A), 34I (B), and J2 (C) were infected with wildtype ETEC strain CVD30 or the CF-deficient mutant strains for 4hrs. Monolayers were washed and lysed to quantify adherent bacteria as % of initial inoculum (CFU recovery). Data presented are pooled from four independent experiments (A, B) and two independent experiments (C). Each dot represents data collected from an individual monolayer. Error bars indicate standard deviations from the means. The asterisks above the bars indicate statistically significant differences determined using one-way ANOVA with Bonferroni’s multiple-comparison test. **, p<0.01; ***, p<0.001; ****, p<0.0001.
(TIFF)

S4 Fig The inhibition of CFA/I-CS21 ETEC adherence to an additional enteroid monolayer by CFA/I-specific, but not CS21-specific, antibodies. Differentiated jejunal enteroid monolayers (J2) were infected with wildtype ETEC strain CVD30 for 4 hrs with or without pre-incubation with anti-CFA/I or anti-CS21 antibody. Monolayers were washed and lysed to quantify adherent bacteria expressed as % of initial inoculum (CFU recovery). Data presented are pooled from two independent experiments. Each dot represents data collected from an individual monolayer. Error bars indicate standard deviations from the means. The asterisks above the bars indicate statistically significant differences determined using one-way ANOVA with Bonferroni’s multiple-comparison test. ***, p<0.001.
(TIFF)

S5 Fig The lack of adherence inhibition by anti-CS21 antibodies in ETEC strains with increased CS21 expression. Differentiated ileal enteroid monolayers (46I) were infected with wildtype ETEC strains E9034A or CVD30 grown in Terrific broth static conditions for 4 hrs with or without pre-incubation with anti-CS21 antibodies. Monolayers were washed and lysed to quantify adherent bacteria expressed as % of initial inoculum (CFU recovery). Data presented are from one independent experiment. Each dot represents data collected from an individual monolayer. Error bars indicate standard deviations from the means. Statistically significant differences were calculated using one-way ANOVA with Bonferroni’s multiple-comparison test.
(TIFF)

S6 Fig The lack of adherence inhibition by normal rabbit sera. Differentiated ileal enteroid monolayers (35I) were infected with wildtype ETEC strain CVD30 for 4 hrs with or without pre-incubation with normal rabbit sera. Monolayers were washed and lysed to quantify adherent bacteria expressed as % of initial inoculum (CFU recovery). Data presented are from one independent experiment. Each dot represents data collected from an individual monolayer. Error bars indicate standard deviations from the means. Statistically significant differences were calculated using unpaired t-test.
(TIFF)

S7 Fig The inhibition of adherence by CFA/I-CS21 ETEC strain isolated from Chile to enteroid monolayers by CFA/I-specific, but not CS21-specific, antibodies. Differentiated ileal enteroid monolayers (46I) were infected with wildtype ETEC strain 10002a from Chile for 4 hrs with or without pre-incubation with anti-CFA/I or anti-CS21 antibody. Monolayers were washed and lysed to quantify adherent bacteria expressed as % of initial inoculum (CFU recovery). Data presented are from one independent experiment. Each dot represents data collected from an individual monolayer. Error bars indicate standard deviations from the means. Statistically significant differences were calculated using one-way ANOVA with Bonferroni’s multiple-comparison test. **, p<0.01.
(TIFF)

S8 Fig ST production by wildtype ETEC strains CVD30 and H10407. ST production was measured by ST-mediated cGMP production in T84 cell monolayers after 8 hr incubation with 1nM ST or ETEC supernatants isolated following growth in DMEM for 8 hrs. Data presented are from one independent experiment. Each dot represents data collected from an individual T84 monolayer. The amount of ST produced by the ETEC isolates was calculated relative to the amount of cGMP produced by the 1nM purified ST positive control with background cGMP (Blank) subtracted. The total amount of ST (ng) was calculated from the ST concentration (nM) using molecular weight of ST. Error bars indicate standard deviations from the means. Statistically significant differences were calculated using unpaired t-test. ****, p<0.0001.
(TIFF)

S9 Fig The polarized production of cGMP in additional enteroid monolayers following purified toxin exposure or infection with CFA/I-CS21 ETEC. Differentiated jejunal enteroid monolayers (J2) were exposed to purified ST toxin (1nM) or infected with wildtype strain CVD30 or its derivative CFA/I-deficient strains for 6 hrs. Apical and basolateral supernatants were collected for cGMP ELISA. Data presented are from one independent experiment. Each dot represents data collected from an individual monolayer. Error bars indicate standard deviations from the means. The asterisks above the bars indicate statistically significant differences determined using one-way ANOVA with Bonferroni’s multiple-comparison test. **, p<0.01.
(TIFF)

S10 Fig Changes in transepithelial electrical resistance (TEER) of additional enteroid monolayers following infection with wildtype or CF-deficient strains. TEER values of differentiated ileal enteroid monolayers (46I) were recorded prior to infection and after 4 hr incubation. The change in TEER is expressed as TEER percentage change. Data presented are from four independent experiments. Each dot represents data collected from an individual monolayer. The asterisks above the dots indicate statistically significant differences determined using one-way ANOVA with Bonferroni’s multiple-comparison test, compared to the TEER of the uninfected enteroid monolayers (Blank). *, p<0.05; **, p<0.01; ***, p<0.001.
(TIFF)
