# Supplementary figures and images for: The role of CFA/I in adherence and toxin delivery by ETEC expressing multiple colonization factors in the human enteroid model
Source: PLoS Negl Trop Dis. 2022 Jul 26;16(7):e0010638. doi: 10.1371/journal.pntd.0010638 (PMC9355178; doi:10.1371/journal.pntd.0010638)

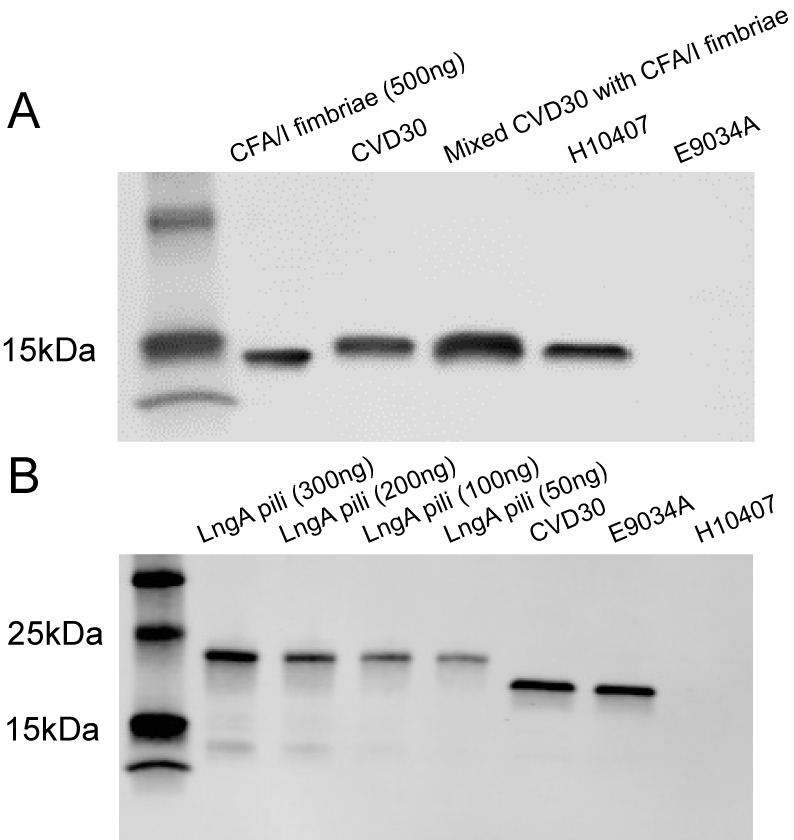

Supplement: S1 Fig — Purified CFA/I fimbriae were run alone and mixed with whole cell lysate alongside whole cell lysates of wildtype strains CVD30, H10407, and E9034A grown on CFA agar. The blot was probed with anti-CFA/I antibodies (A). Purified CFA/I results in a CfaB band that migrates faster than that in whole cell lysates. Purified LngA peptide was titrated and whole cell lysates of wildtype strains CVD30, E9034A, and H10407 grown in Terrific broth static conditions overnight were run on a western blot probed with anti-CS21 antibodies (B). Purified LngA is tagged by a 6X His tag (21kDa) while LngA in whole cell lysates result in a band at 19kDa. (TIFF) [file pntd.0010638.s001.tiff]

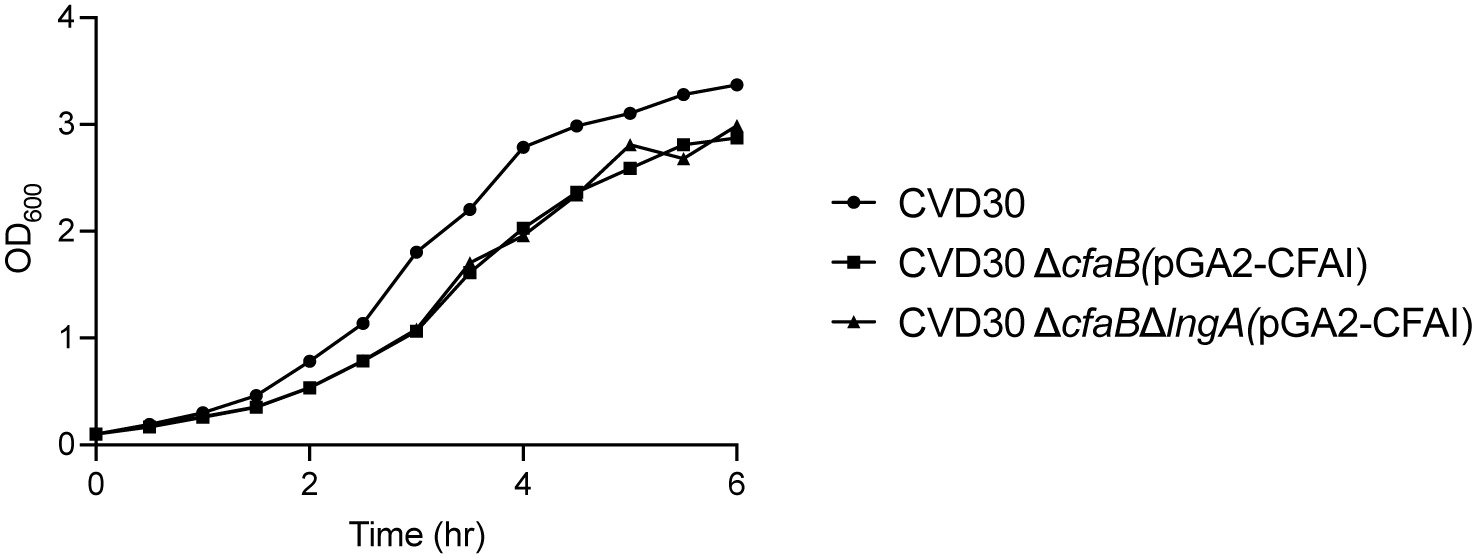

Supplement: S2 Fig — Growth curve of ETEC strains CVD30 and complemented mutants CVD30ΔcfaB(pGA2-CFA/I) and CVD30ΔcfaBΔlngA(pGA2-CFA/I) was assessed at 37°C with shaking in DMEM. Growth was monitored every 30 min by measuring OD600. (TIFF) [file pntd.0010638.s002.tiff]

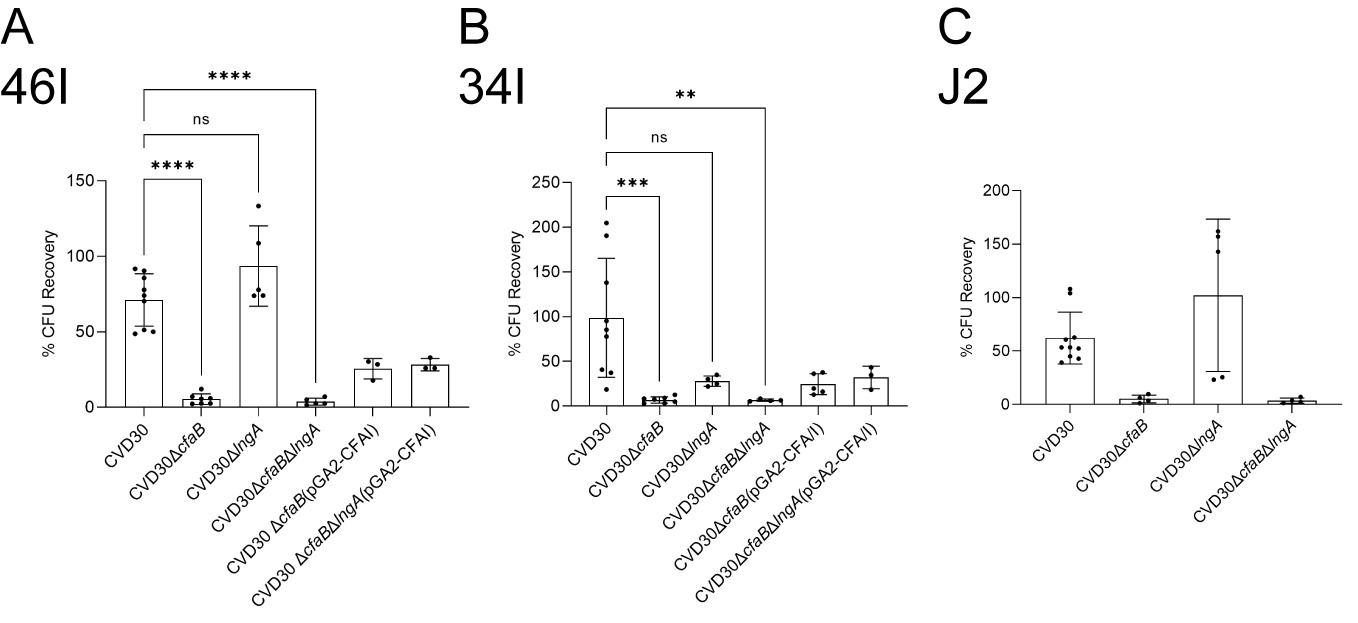

Supplement: S3 Fig — Differentiated jejunal and ileal enteroid monolayers 46I (A), 34I (B), and J2 (C) were infected with wildtype ETEC strain CVD30 or the CF-deficient mutant strains for 4hrs. Monolayers were washed and lysed to quantify adherent bacteria as % of initial inoculum (CFU recovery). Data presented are pooled from four independent experiments (A, B) and two independent experiments (C). Each dot represents data collected from an individual monolayer. Error bars indicate standard deviations from the means. The asterisks above the bars indicate statistically significant differences determined using one-way ANOVA with Bonferroni’s multiple-comparison test. **, p<0.01; ***, p<0.001; ****, p<0.0001. (TIFF) [file pntd.0010638.s003.tiff]

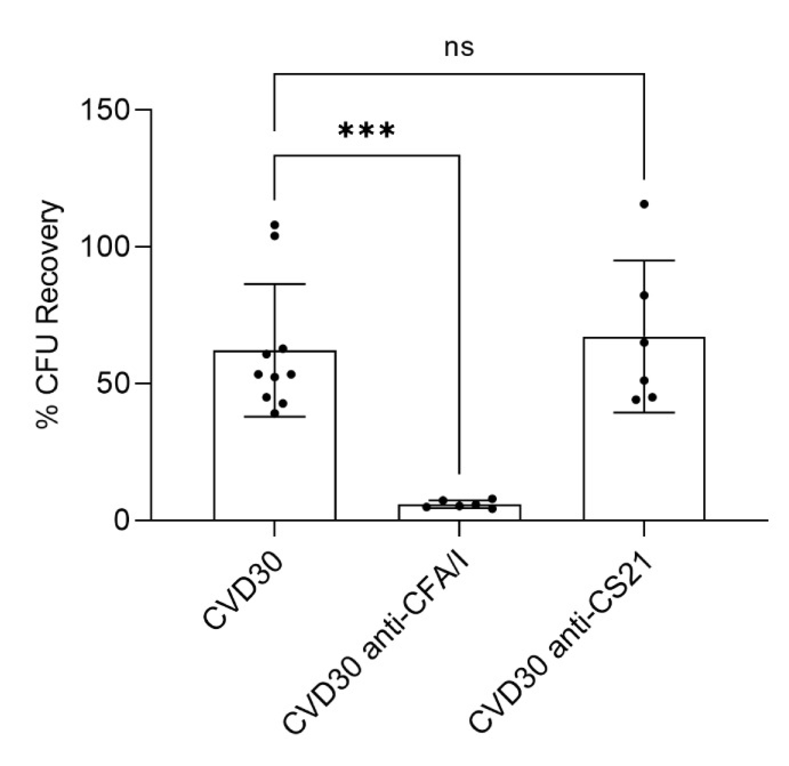

Supplement: S4 Fig — Differentiated jejunal enteroid monolayers (J2) were infected with wildtype ETEC strain CVD30 for 4 hrs with or without pre-incubation with anti-CFA/I or anti-CS21 antibody. Monolayers were washed and lysed to quantify adherent bacteria expressed as % of initial inoculum (CFU recovery). Data presented are pooled from two independent experiments. Each dot represents data collected from an individual monolayer. Error bars indicate standard deviations from the means. The asterisks above the bars indicate statistically significant differences determined using one-way ANOVA with Bonferroni’s multiple-comparison test. ***, p<0.001. (TIFF) [file pntd.0010638.s004.tiff]

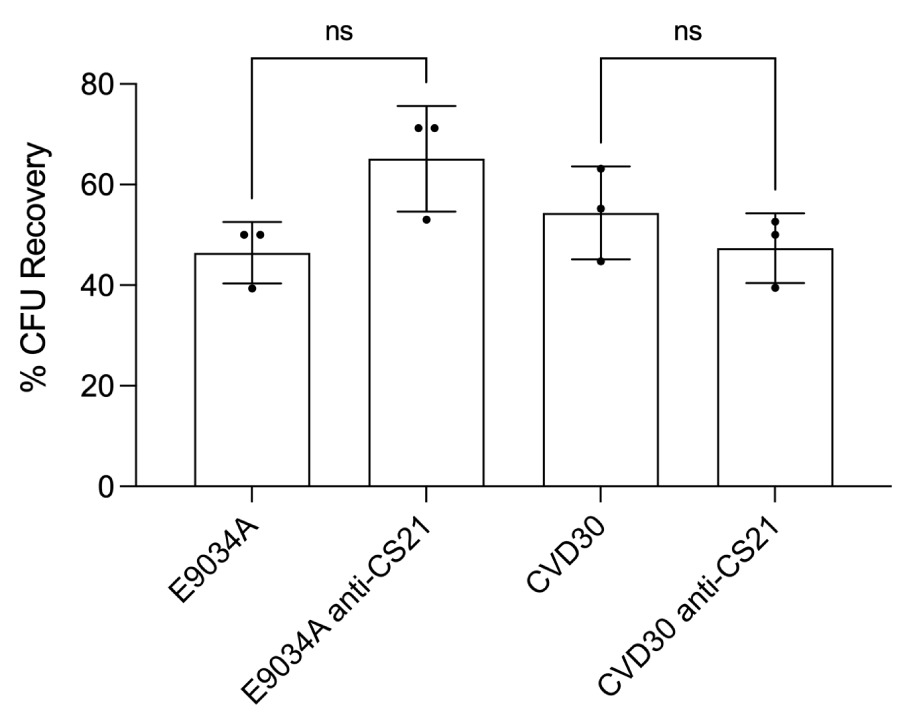

Supplement: S5 Fig — Differentiated ileal enteroid monolayers (46I) were infected with wildtype ETEC strains E9034A or CVD30 grown in Terrific broth static conditions for 4 hrs with or without pre-incubation with anti-CS21 antibodies. Monolayers were washed and lysed to quantify adherent bacteria expressed as % of initial inoculum (CFU recovery). Data presented are from one independent experiment. Each dot represents data collected from an individual monolayer. Error bars indicate standard deviations from the means. Statistically significant differences were calculated using one-way ANOVA with Bonferroni’s multiple-comparison test. (TIFF) [file pntd.0010638.s005.tiff]

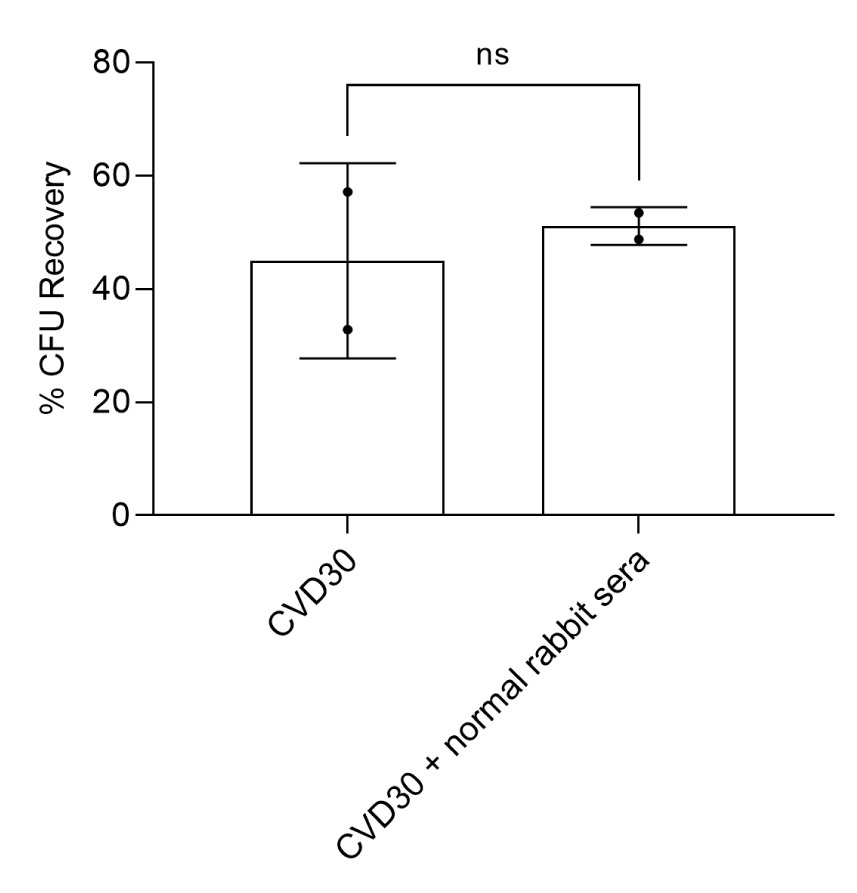

Supplement: S6 Fig — Differentiated ileal enteroid monolayers (35I) were infected with wildtype ETEC strain CVD30 for 4 hrs with or without pre-incubation with normal rabbit sera. Monolayers were washed and lysed to quantify adherent bacteria expressed as % of initial inoculum (CFU recovery). Data presented are from one independent experiment. Each dot represents data collected from an individual monolayer. Error bars indicate standard deviations from the means. Statistically significant differences were calculated using unpaired t-test. (TIFF) [file pntd.0010638.s006.tiff]

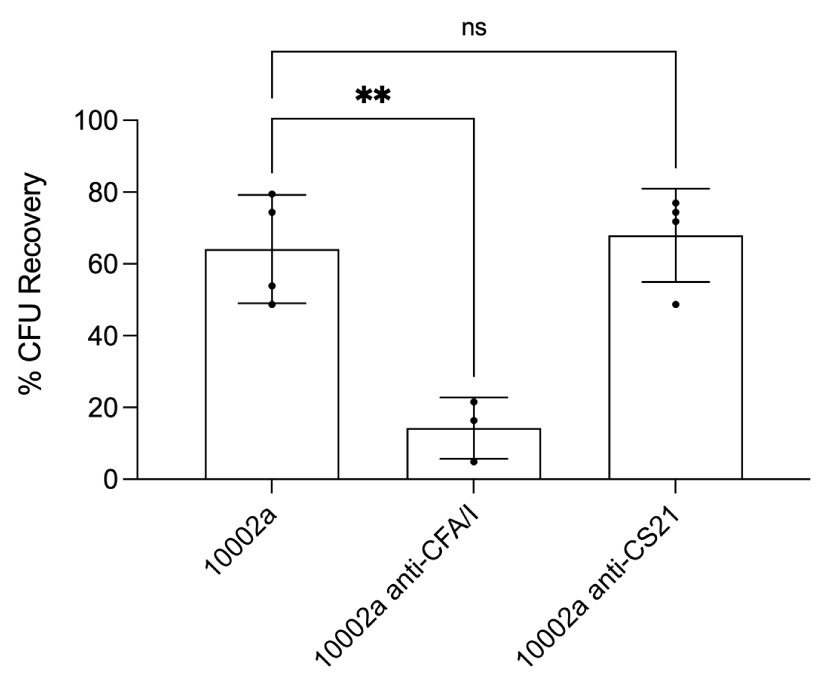

Supplement: S7 Fig — Differentiated ileal enteroid monolayers (46I) were infected with wildtype ETEC strain 10002a from Chile for 4 hrs with or without pre-incubation with anti-CFA/I or anti-CS21 antibody. Monolayers were washed and lysed to quantify adherent bacteria expressed as % of initial inoculum (CFU recovery). Data presented are from one independent experiment. Each dot represents data collected from an individual monolayer. Error bars indicate standard deviations from the means. Statistically significant differences were calculated using one-way ANOVA with Bonferroni’s multiple-comparison test. **, p<0.01. (TIFF) [file pntd.0010638.s007.tiff]

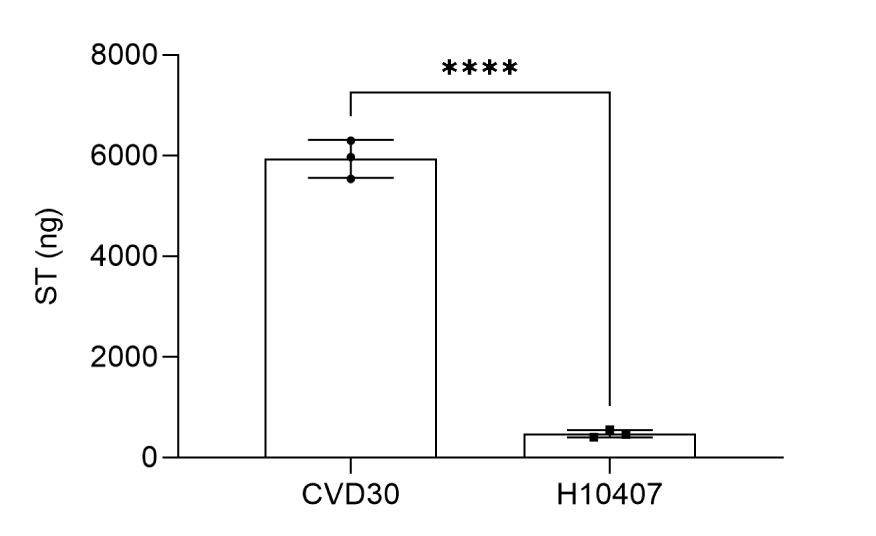

Supplement: S8 Fig — ST production was measured by ST-mediated cGMP production in T84 cell monolayers after 8 hr incubation with 1nM ST or ETEC supernatants isolated following growth in DMEM for 8 hrs. Data presented are from one independent experiment. Each dot represents data collected from an individual T84 monolayer. The amount of ST produced by the ETEC isolates was calculated relative to the amount of cGMP produced by the 1nM purified ST positive control with background cGMP (Blank) subtracted. The total amount of ST (ng) was calculated from the ST concentration (nM) using molecular weight of ST. Error bars indicate standard deviations from the means. Statistically significant differences were calculated using unpaired t-test. ****, p<0.0001. (TIFF) [file pntd.0010638.s008.tiff]

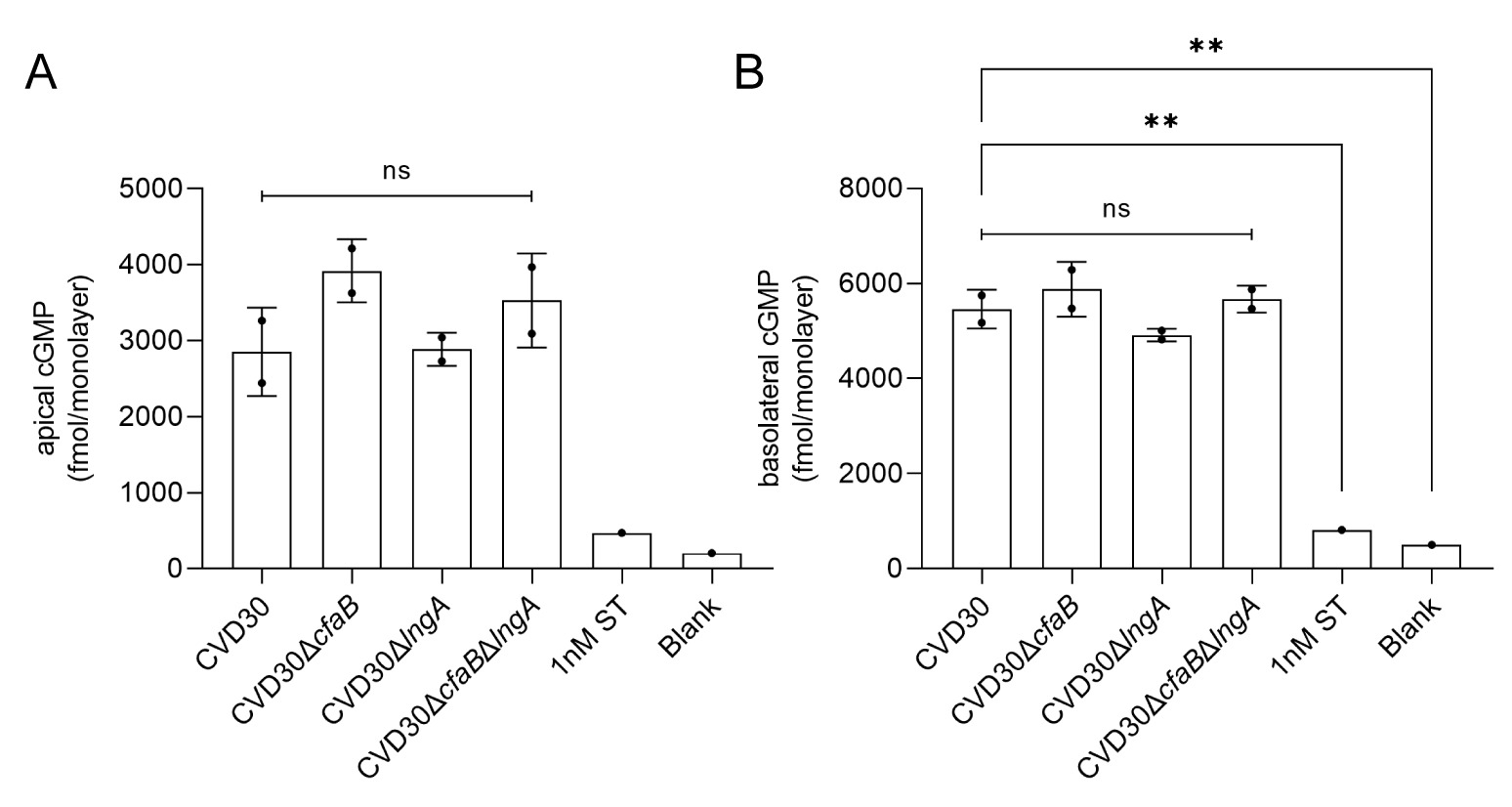

Supplement: S9 Fig — Differentiated jejunal enteroid monolayers (J2) were exposed to purified ST toxin (1nM) or infected with wildtype strain CVD30 or its derivative CFA/I-deficient strains for 6 hrs. Apical and basolateral supernatants were collected for cGMP ELISA. Data presented are from one independent experiment. Each dot represents data collected from an individual monolayer. Error bars indicate standard deviations from the means. The asterisks above the bars indicate statistically significant differences determined using one-way ANOVA with Bonferroni’s multiple-comparison test. **, p<0.01. (TIFF) [file pntd.0010638.s009.tiff]

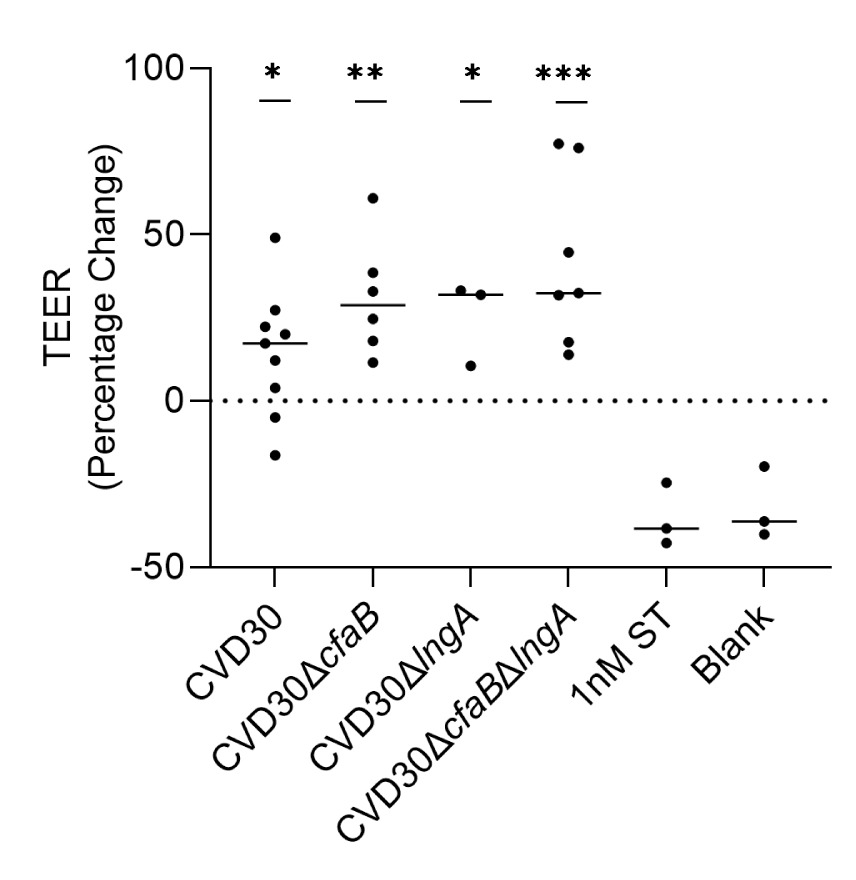

Supplement: S10 Fig — TEER values of differentiated ileal enteroid monolayers (46I) were recorded prior to infection and after 4 hr incubation. The change in TEER is expressed as TEER percentage change. Data presented are from four independent experiments. Each dot represents data collected from an individual monolayer. The asterisks above the dots indicate statistically significant differences determined using one-way ANOVA with Bonferroni’s multiple-comparison test, compared to the TEER of the uninfected enteroid monolayers (Blank). *, p<0.05; **, p<0.01; ***, p<0.001. (TIFF) [file pntd.0010638.s010.tiff]
